# Enhancing corrosion resistance with chemically modified aluminum oxide in UV-curable coatings applied to steel surfaces

**DOI:** 10.1038/s41598-025-99898-6

**Published:** 2025-05-14

**Authors:** M. Attia, Mahmoud Basseem I. Mohamed, M. A. Hegazy, M. M. Ghobashy, H. Abd El-Wahab, F. Abdelhai

**Affiliations:** 1 Chemistry Department, Faculty of Science, Al-Azhar University, P.O. 11884, Nasr City, Cairo, Egypt; 2 Petrochemicals Department, Egyptian Petroleum Research Institute (EPRI), Nasr City, Cairo, 11727 Egypt; 3 Radiation Research of Polymer Chemistry Department, National Center for Radiation Research and Technology (NCRRT), Atomic Energy Authority, P.O. Box 8029, Nasr City, Cairo, Egypt

**Keywords:** UV-curable coatings, Corrosion resistance, Nanocomposite, Environmental sustainability, Epoxy acrylate resin, Silane coupling agents, Origin of life, Polymer chemistry, Surface chemistry

## Abstract

This study introduces a novel, environmentally sustainable epoxidized soybean oil acrylate (ESOA) nanocomposite coating containing nAl_2_O_3_-silane nanoparticles (ESOA@TMPTA-nAl_2_O_3_-Silane), which was fabricated using ultraviolet (UV) curing technology. As far as we know, this is the first study to incorporate aluminum oxide nanoparticles (nAl_2_O_3_) modified through covalent bonding with a reactive diluent monomer, tripropylene glycol diacrylate (TPGDA), and a coupling agent to enhance their dispersibility and interaction within the polymer matrix. Comprehensive characterization techniques, including Fourier-transform infrared spectroscopy (FTIR), scanning electron microscopy (SEM), atomic force microscopy (AFM), X-ray diffraction (XRD), UV-spectroscopy, energy-dispersive X-ray spectroscopy (EDX), and transmission electron microscopy (TEM), confirmed the nanocomposite’s structural and polymer morphological enhancements. Electrochemical impedance spectroscopy (EIS) demonstrated a substantial increase in polarization resistance (*R*_p_), rising from 25.6 kΩ cm^2^ for the unmodified polymer to 288.7 kΩ cm^2^ upon the incorporation of (8 wt%) nAl_2_O_3_-Silane. In a similar vein, Potentiodynamic polarization (PDP) exhibited a significant decrease in corrosion current density (*i*_corr_), diminishing from 0.82 to 0.059 µA/cm^2^, thereby achieving an inhibition efficiency exceeding 99%. Additionally, the salt spray test data showed a considerable improvement in the rust degree from 3 to 8G under identical conditions. The data demonstrates the outstanding corrosion resistance characteristics that the nAl_2_O_3_-Silane nanoparticles provided when coupled with the steel substrate. This improvement is attributed to the excellent dispersion, excellent barrier properties, transparency of the resulting coatings and strong adhesion of nAl_2_O_3_-Silane dispersed in the polymer matrix.

## Introduction

Metal corrosion is a pervasive and unavoidable issue that presents substantial security risks and financial setbacks for contemporary society and industrial manufacturing^1,2^. Metal corrosion is predominantly initiated by the chemical reaction between corrosive substances and steel materials^3^. Consequently, avoiding direct contact between steel components and corrosive substances is critical for preventing corrosion damage. Inhibiting the formation of corrosion-reinforced materials has become notably easier and more prevalent with the advent of polymer composite coatings^4,5^.

A considerable proportion of current polymer composite coatings are established through heat-curing procedures and systems based on organic solvents. These methods result in the emission of detrimental volatile organic compounds (VOCs) and over energy consumption^6^. This practice gives rise to substantial environmental contamination and presents possible risks to human well-being^7^. In extreme caution, numerous nations have implemented stringent policies and regulations that target (VOC) emissions^8^. As a result, there has been a renewed emphasis on the advancement of environmentally sustainable surface protective coatings that perform well in shielding and emitting minimal (VOC) emissions^9^. UV-curing technology has become increasingly popular due to its manifold advantages, which include expeditious reaction rates, curing without the need for solvents at room temperature, minimal energy demands, and an economically viable configuration^7^. This technology is essential in adhesives, printing ink, coatings, and surface modification processes^10^. It finds diverse applications in electronics, printing, optics, and electro-optical materials. Significantly, in recent times, the application of UV-curing technology has broadened to encompass the assembly of various electronic devices, where it serves as a crucial component in carrying out processes such as encapsulation, adhesion, and protective measures^11^. Combining the advantages of epoxy resin and acrylate, epoxy acrylate exhibits an extensive array of qualities^12^. These include exceptional resistance to solvents and chemicals, resilience to diverse weather conditions, increased flexibility, exceptional resistance to wear, strong adhesion to substrates, and the capability to fine-tune performance attributes.

In order to improve the protective properties of coatings and address the stringent demands of a wide range of applications in challenging working environments, barrier additives have been integrated into polymer coatings to function as corrosion-resistant constituents. The additives above consist of manganese oxide, silicon oxide, alumina (Al_2_O_3_), montmorillonite, and zinc oxide, among others^13–17^. Alumina is a notable two-dimensional material characterized by its distinctive structure, which grants it exceptional thermal, optical, electrical, catalytic, and mechanical properties^18^. UV-curable coatings often lack strong barrier properties against water, oxygen, and corrosive ions^19^, as well as advanced functionalities like self-healing or enhanced mechanical strength, which are crucial for steel protection. Adding materials, such as Aluminum oxide (Al_2_O_3_) nanoparticles create a dense and tortuous diffusion path within the coating, reducing the penetration of water, oxygen, and corrosive ions like chloride, which are critical factors in corrosion. Al_2_O_3_ nanoparticles enhance the mechanical properties of the UV-curable coating, improving its resistance to wear, scratches, and mechanical stress that could expose the substrate to corrosive environments^20,21^. Li et al.^22^ developed a novel UV-curable coating for corrosion resistance and coated shielding, incorporating molybdic acid and cerium ions. The UV-curable resin in this study formed strong chemical bonds with the metal substrate, significantly enhancing the coating’s mechanical properties. Similarly, a bio-epoxy thermoset derived from epoxidized soybean was designed as an anti-corrosion coating to protect carbon steel structures in harsh marine environments^23^. Furthermore, the use of an epoxy silane coupling agent for molybdenum disulfide enables an effective embedment of the nanoparticles into the epoxy matrix, enhancing the coating’s corrosion resistance^24^. It is well known from the literature that Al_2_O_3_ nanoparticles exhibit a significant absorption peak at 255 nm due to photoexcitation of electrons from the valence to conduction band^25,26^. Thus, the role of Al_2_O_3_ nanoparticles in the curing process can be explained as follows: these nanoparticles absorb UV radiation, leading to the photoexcitation of electrons in the valence band. The excited electrons then initiate the curing process. Additionally, Al_2_O_3_ nanoparticles contribute to enhancing the corrosion resistance of epoxy coatings^27^, and provide antibacterial protection^28^, Furthermore, the fabrication of aluminum oxide nanoparticles using graphene oxide has been explored to improve the anti-corrosion performance of composite epoxy coatings^29^^,^^30^.

In this context, this study deals with the preparation of aluminum oxide (Al_2_O_3_) nanoparticles via the sol–gel method, leveraging their exceptional mechanical and thermal stability to enhance the curing and protective performance of the resultant coating. To further improve their dispersion and interfacial compatibility, Al_2_O_3_ nanoparticles were surface-modified with γ-Glycidoxy propyl trimethoxy silane and subsequently dispersed in tripropylene glycol diacrylate monomer using an ultrasonic homogenizer, yielding a stable dispersion of spherical (nAl_2_O_3_-Silane) nanoparticles. To develop a sustainable and UV-curable epoxy acrylate coating, epoxidized soybean oil acrylate (ESOA) and trimethylol propane triacrylate (TMPTA) were incorporated as renewable and environmentally friendly components. The prepared nAl_2_O_3_-Silane nanoparticles dispersion was then introduced into the epoxy acrylate matrix to initiate the curing process and form a polymer nanocomposite coating with enhanced protective properties.

A comprehensive characterization of the prepared materials coatings has been thoroughly investigated. The corrosion resistance and durability of the nanocomposite coatings were evaluated through electrochemical measurements, neutral salt spray tests, as well as mechanical and physical property assessments respectively. Our findings are significant because it maintains the final coating’s transparency despite the presence of nA_2_lO_3_-silane nanoparticles. These findings differ from previous research in terms of transparency, as most polymer nanocomposites are translucent or opaque. According to our findings, the final coating can be effectively used to protect metal-based artifacts against corrosion since they provide a transparent coating layer.

## Experimental

### Materials

Epoxidized soya bean oil acrylate (ESOA), Trimethylol propane triacrylate (TMPTA), and tripropylene glycol diacrylate (TPGDA) were supplied by Allnex GmbH. Aluminum nitrate (Al_2_(NO_3_)_3_·9H_2_O) 98%, citric acid 99.5%, ethanol absolute, hydrochloric acid 36%, and p-toluene sulfonic acid 98.5% were obtained from Across Co. γ-Glycidoxy propyl trimethoxy silane (GPTMS) 98%, used as a coupling agent and surface treatment, was supplied by Dow Chemical Company. Disperbyk 163, a dispersing agent, was provided by BYK GmbH. Benzophenone 99.7%, the photoinitiator, was sourced from TCI, United States of America.

### Synthesis of Al_2_O_3_ nanoparticles

The synthesis of aluminum oxide (Al_2_O_3_) nanoparticles was achieved using the sol–gel method. Initially, a solution comprising aluminum nitrate and citric acid in deionized water was prepared. During the process, the molar ratio of aluminum nitrate to citric acid was kept at 1:1, and the solution was heated at 60 °C, resulting in a yellowish solution. Subsequently, the temperature was raised to 80 °C, forming a transparent, adhesive gel. This gel underwent a process where it was subjected to 2 h of heat treatment at 200 °C, yielding a polymeric citrate precursor. Finally, the precursor was calcined at 900 °C for 2 h, forming the Al_2_O_3_ nanoparticles^31,32^.

### Surface modification and dispersion of nAl_2_O_3_

Surface modification of aluminum oxide nanoparticles (nAl_2_O_3_) was carried out by first hydrolyzing the glycidoxy propyl trimethoxy silane (GPTMS) precursor. Three trials were carried out utilizing varying GPTMS concentrations (2, 5, and 10 g) in an ethanol–water mixture (80:20 v/v) have been made to reach the optimum conditions. The pH was adjusted to 2 using conc. hydrochloric acid (HCl), and the resulted solution was re-fluxed for 1 h. The storage stability was tested over a 20-day period, and the 5 g sample had the best stability, with no sedimentation or separation, suggesting that it was the appropriate concentration.

**Fig. 1:**
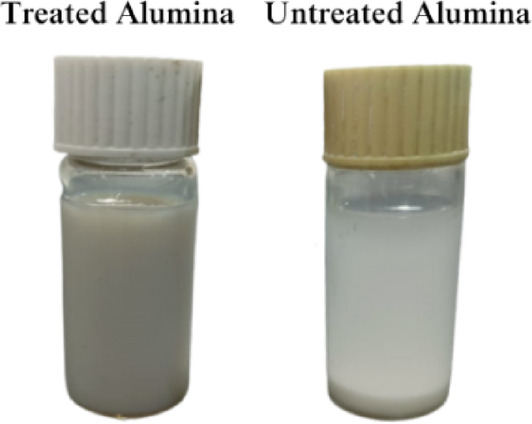
Two samples of dispersion treated and untreated nAl_2_O_3_ after 20 days.

Furthermore, the alumina powder was hydrated in deionized water and dried. A portion of the hydrated alumina (5 g) was treated with 250 ml of the hydrolyzed GPTMS solution of 5 g concentration to provide optimal surface coverage without excessive aggregation. The mixture was then refluxed at 80 °C with p-toluene sulfonic acid (0.1 g) as a catalyst for 3 h to facilitate surface salinization. Afterward, the modified alumina was centrifuged, rinsed with toluene and methanol, and then dried in an oven at 80 °C for 12 h^33–35^. The surface-treated alumina was then dispersed in a mixture of tripropylene glycol diacrylate (TPGDA) monomer and Disperbyk 163, using an ultrasonic homogenizer while cooling to below 35 °C. This dispersion remained stable for 20 days without showing any signs of gelation or sedimentation, indicating the successful dispersion of the surface-treated alumina nanoparticles, as shown in Fig. 1.

### Coating film preparation and curing

UV-curable formulations (A–E) were prepared by combining epoxy soybean oil acrylate (ESOA), trimethylol propane triacrylate (TMPTA), and benzophenone with varying concentrations of surface-treated aluminum oxide nanoparticles (nAl_2_O_3_-Silane) as outlined in Table 1. These formulations were then degassed to remove air bubbles, applied onto steel panels by film applicator to achieve uniform coating thickness, and cured using ultraviolet (UV) irradiation for 20 s. The UV curing process utilized a mercury lamp with an intensity ranging from 80 to 120 W/cm^2^, manufactured by T_max_, China. This lamp emitted light within the wavelength range of 340 to 360 nm, facilitating the curing process of the coatings.

**Table 1:** UV-curable formulations (**A-E**) at different ratio of constituents.

Components (wt%)	Formulations				
	A	B	C	D	E
Epoxidized soya been acrylate	50	50	50	50	50
TMPTA	44	44	44	44	44
Benzophenone	6	6	6	6	6
Treatment nAl_2_O_3_-Silane dispersion in TPGDA	0	2	4	6	8

### Instruments

The chemical composition of the prepared polymers was analyzed using an FT-IR Spectrometer (Thermo-Fischer Nicolet™ iS™ 10, USA) with a resolution of 1 cm^–1^ over a wavenumber range of 400–4000 cm^–1^. The Beckman DU 7400 spectrophotometer was utilized to perform UV–visible spectrophotometry. For SEM analysis (Tescan VEGA3), an ultrathin gold layer was applied to samples to investigate nanoparticle morphology and dispersion via TEM (JEM-2100 plus). Elemental analysis by EDX confirmed the existence of Al and Si. X-ray diffraction (XRD) was employed to determine the phase structure and crystallinity of the substances via an. XRD-7000 (Schi-madzu, Germany), images of surface topographies were recorded by using atomic force microscope (AFM model Wet–SPM (Scanning Probe microscope) Shimadzu, Japan. The surface hydrophobicity of the UV-cured polymers was probed by the sessile drop method, using the DMO 601 device from Japan. A refractometer from Mettler Toledo obtained the refractive index value, is an analytical instrument that uses a high-resolution optical sensor to measure the total reflection of a light beam that is in contact with a sample. The haze measurement of the coating composites was conducted using an X-Rite Ci7600 spectrophotometer in accordance with ASTM D1003. Electrochemical impedance spectroscopy (EIS) and Tafel polarization experiments were employed to evaluate the resistance of solutions using an Origa flex OGF 500.

#### Chemical composition of carbon steel

Tests were performed on carbon steel of the following composition (wt%): 0.19% C, 0.05% Si, 0.94% Mn, 0.009% P, 0.004% S, 0.014% Ni, 0.009% Cr, 0.034% Al, 0.016% V, 0.003% Ti, 0.022% Cu, and the rest Fe.

### Contact angle measurement

The wettability of the UV-cured coatings was determined by the contact angle of water molecules with the coated steel substrates. The surface energy and hydrophobic characteristics of the coatings were revealed.

### Gel fraction

The gel fraction, which determines the crosslinked density of the cured coatings, was received by dipping cured film in acetone and followed by a constant weight drying process. By utilizing Eq. (1), the gel fraction was calculated.

1 $$\text{Gel fraction }\left({\%}\right)=\left(\frac{{W}_{t}}{{W}_{0}}\right)$$

where ${W}_{0}$ is the original weight of the sample before immersion, and ${W}_{t}$ is the weight of the dried sample after immersion.

### Physico-chemical and mechanical properties

The properties of the cured coatings were evaluated using standard test methods. Solvent resistance was assessed via the MEK rub test (ASTM D 4752), which determines the degree of cure based on the film’s resistance to methyl ethyl ketone. Chemical resistance was evaluated by measuring the weight loss (%) of polymers after 12 h immersion in (10%) HCl and (10%) NaOH at room temperature (23 ± 2 °C). The gloss test (ASTM D523) measures the specular reflectance of a surface to evaluate its gloss level at specific angles (60°). The scratch test, conducted in accordance with ASTM D7027, measured the critical load required to initiate a scratch, expressed in newtons (N). Cross cut tape adhesion test (ASTM D3359). Salt spray testing is used for evaluating the performance of metal surfaces in various environments. In this study, carbon steel panels (Q-Panel) were selected as substrates and coated with UV-curable polymers, both with and without the incorporation of nAl_2_O_3_-Silane. The coated panels were then subjected to a neutral salt spray fog test for 500 h under conditions specified by ASTM B117. The extent of corrosion was assessed through visual analysis, following the guidelines of ASTM D610 to determine the degree of rust formation. Electrochemical impedance spectroscopy (EIS) and Tafel polarization experiments were employed to evaluate the resistance of solutions immersed in (3.5 wt%) NaCl by using Origa flex OGF 500. The carbon steel sample (One-centimeter square was exposed) as a working electrode, silver/silver chloride (Ag/AgCl) as a reference electrode, and platinum wire as a counter electrode made up the three-electrode cell design. The open circuit potential (OCP) was finished first to attain stability. Using a 10-mV signal amplitude, electrochemical impedance spectroscopy (EIS) measurements were carried out at the Open Circuit Potential (OCP) over a frequency spectrum spanning from 100 kHz to 10 mHz. ZSimpwin software analyzed the EIS data using a particular equivalent circuit model. Dynamic polarization (DP) measurements were conducted over a voltage range of ± 200 mV in relation to the OCP, with a scanning rate of 0.2 mV/s.

## Results and discussion

### Synthesis

#### Synthesis of ESOA@TMPTA polymer

**Fig. 2:**
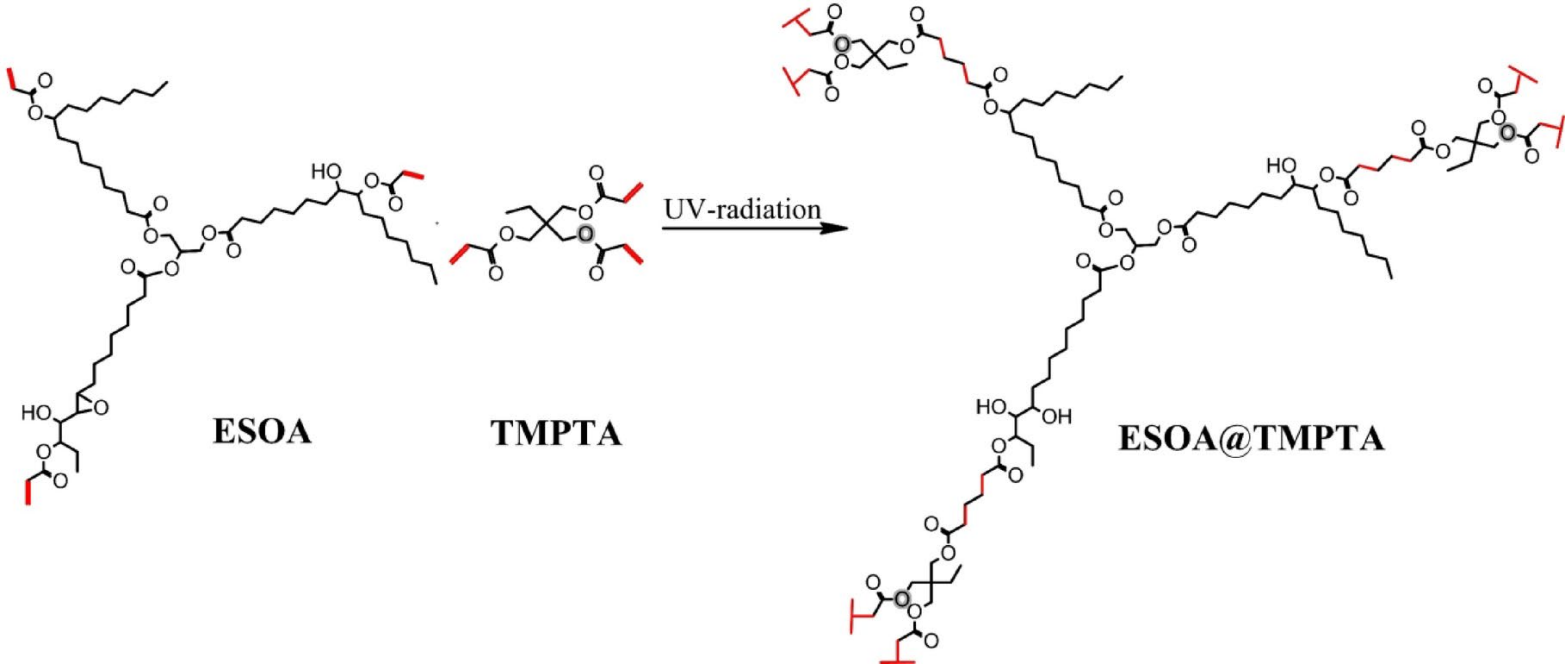
Synthesis of ESOA@TMPTA polymer.

The synthesis of the ESOA@TMPTA polymer is an essential component in advancing our innovative nanocomposite coating. The synthesis was obtained by reacting the ESOA and TMPTA to afford polymer, as shown in Fig. 2.

ESOA was combined with TMPTA in a predetermined proportion to attain an ideal equilibrium between flexibility and mechanical strength. The rationale for choosing TMPTA, a multifunctional acrylate monomer, was its capacity to generate a solid, three-dimensional crosslinked structure, which substantially improved the physical attributes of the polymer. The ESOA@TMPTA underwent ultraviolet curing with the assistance of a photoinitiator to accelerate the polymerization process.

**Fig. 3:**
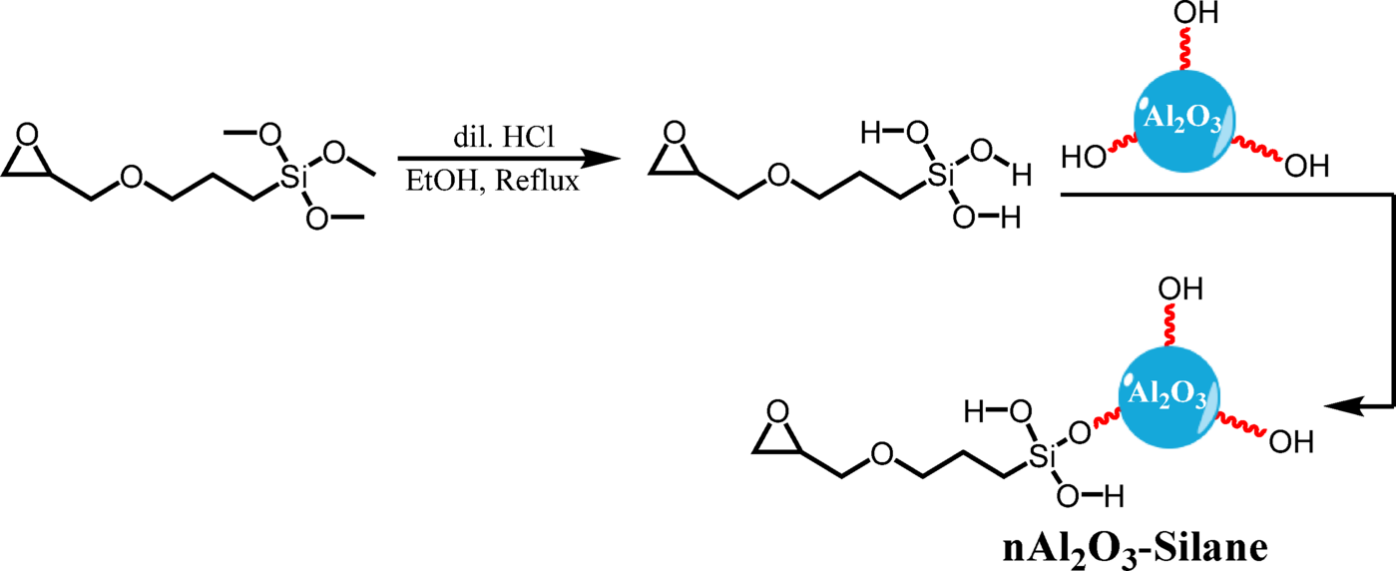
Synthesis of nAl_2_O_3_- Silane.

In order to fully activate the surface with hydroxyl (–OH) groups, the nAl_2_O_3_-Silane were intentionally hydroxylated^36^. The hydroxylated surface plays a critical role in facilitating the ensuing reaction with the silane hydroxy groups, thereby enabling strong chemical bonds to be formed as shown in Fig. 3.

**Fig. 4:**
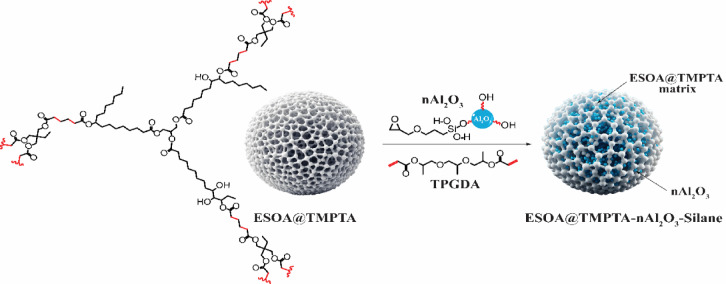
Synthesis of ESOA@TMPTA-nAl_2_O_3_-Silane.

The synthesis of the ESOA@TMPTA-nAl_2_O_3_-Silane was conducted with great attention to detail to capitalize on the distinctive characteristics of TMPTA@ESOA, while also incorporating the increased robustness offered by nAl_2_O_3_-Silane. The procedure commenced with formulating the reactive polymer matrix, which consisted of a mixture of ESOA and TMPTA. Surface-modified nanoparticles (nAl_2_O_3_-Silane) were incorporated gradually into this matrix, guaranteeing consistent distribution throughout the polymer blend as shown in Fig. 4.

It was essential to modify the surface of nAl_2_O_3_ before incorporating them into the composite to improve their compatibility with the organic matrix and promote a more uniform distribution of the nanoparticles. In this stage, the nAl_2_O_3_ were treated with γ-glycidoxy propyl trimethoxy silane (GPTMS). This treatment introduced functional groups into the nanoparticles, which formed stronger bonds with the ESOA and TMPTA. As a result, aggregation was reduced, and the mechanical and protective properties of the polymer nanocomposite were enhanced.

After the alterations were made to nAl_2_O_3_-Silane, the resulting solution was exposed to UV radiation to commence the curing procedure.

### Characterization of the ESOA@TMPTA-nAl_2_O_3_-silane nanocomposite coating

#### Spectroscopic analysis

##### Fourier transform infrared spectroscopy (FTIR)

**Fig. 5:**
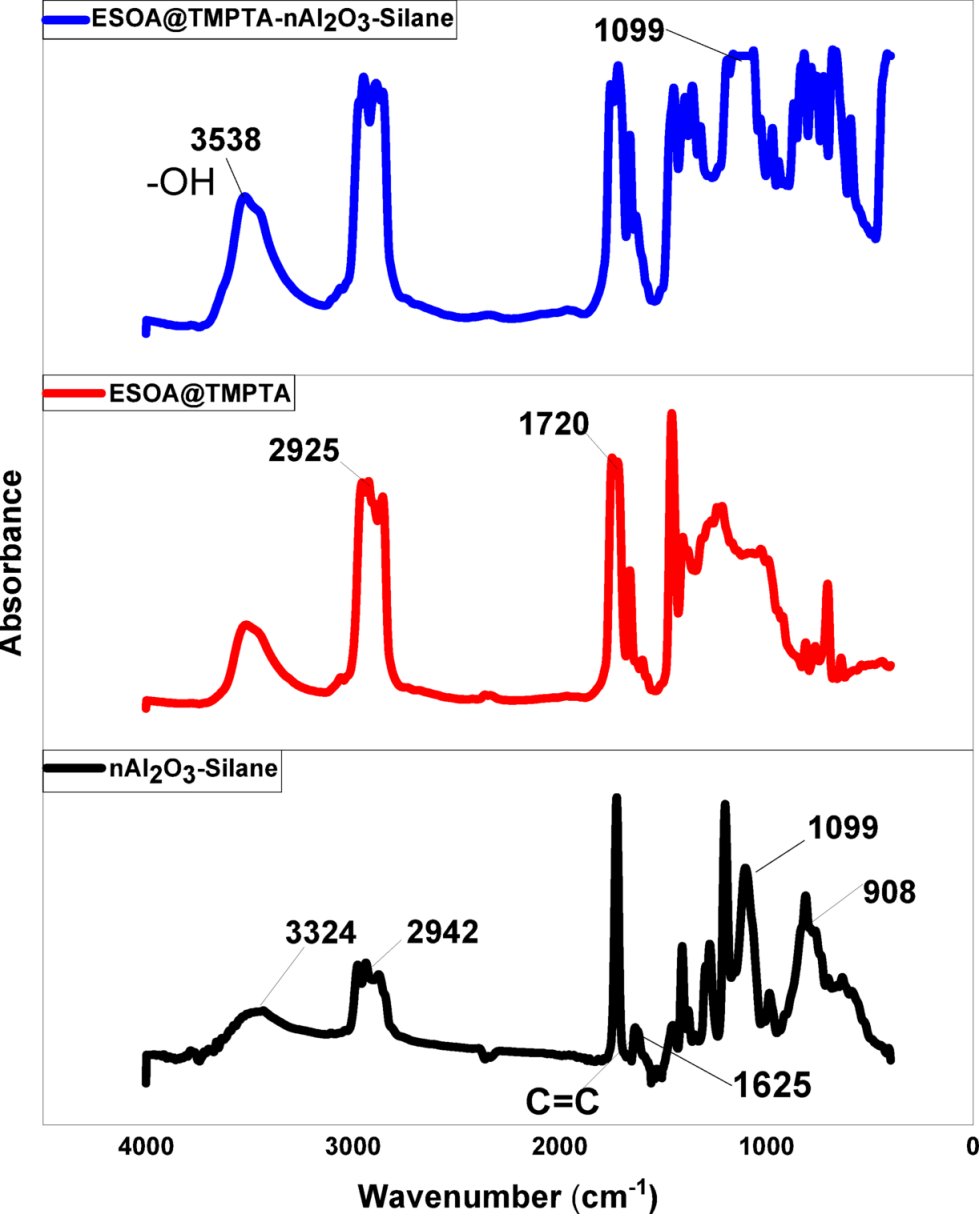
IR spectra of nAl_2_O_3_-Silane, ESOA@TMPTA, and ESOA@TMPTA-nAl_2_O_3_-Silane.

This analysis identified silane coupling agents based on Al–O–Si bonds in nAl_2_O_3_-Silane (approximately 1100 cm^−1^)^37^, and C–H stretching of silane coupling agents (approximately 2940 cm^−1^)^34^. The strong peak of the surface hydroxyl group was shifted toward the lower wavenumber region at 3324 cm^−1^ in the FTIR spectra of surface-modified nano-alumina. This type of shifting was due to the effective interaction between the surface hydroxyl group of nano alumina and silane coupling agents^38^, and the epoxy band at 908 cm^−1^ and 1625 cm^−1^ correspond to (C=C) in TPGDA. Therefore, FTIR results suggested that the treatment of silane coupling agent successfully modified the surface hydroxyl groups of nano-alumina. The FTIR spectra for UV-cured polymer has a characteristic peak located at 1720 cm^−1^, corresponding to carbonyl group C=O, FTIR peaks centered at 2860 cm^−1^ and 2925 cm^−1^ corresponding to asymmetric and symmetric stretching of aliphatic C–H, respectively, A peak at 3247–3538 cm^1^ corresponds to the hydroxyl group (–OH), and a peak at 1240 cm^–1^ corresponds to the (–C–O) group based on the epoxide ring opening in epoxidized soybean acrylate oligomer. In the UV-cured formulation, with and without nanoparticles (Fig. 5) confirms successful curing^39,40^.

##### UV–visible spectroscopy

**Fig. 6:**
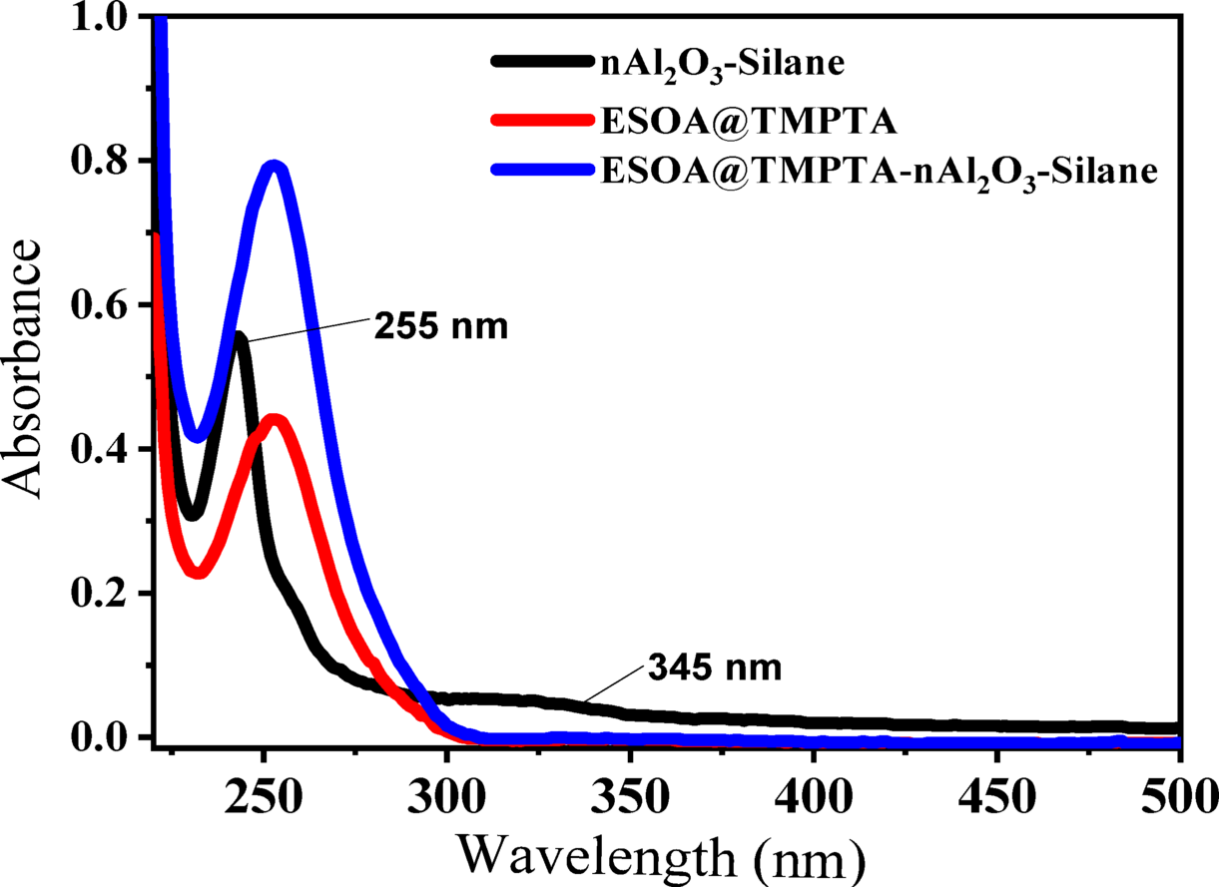
UV–Vis spectra of nAl_2_O_3_-Silane, ESOA@TMPTA, and ESOA@TMPTA-nAl_2_O_3_-Silane.

UV–visible spectroscopy analysis was conducted on UV-cured polymer with and without the addition of surface-treated aluminum oxide nanoparticles (nAl_2_O_3_-Silane) and the dispersion of nAl_2_O_3_-Silane. The results depicted in Fig. 6 indicate that the UV-cured polymer, regardless of the presence of nAl_2_O_3_-Silane, exhibits similar visible spectral features to their respective parent compounds. Notably, the absorption peak was observed at wavelengths of 255 nm, attributed to the (n-π*) of carbonyl group of the polymer molecule. A peak that appears at 345 nm (n-σ*) indicates that there is a slight difference between the dispersion of nAl_2_O_3_-Silane and the polymer composite. This difference is attributed to the epoxy group in the coupling agent. The UV–Vis absorption spectra of nAl_2_O_3_-Silane shows an excitation band centered at 255 nm. The band at 255 nm is often reported^25^, and it corresponds to an undoped oxide with defects caused by oxygen vacancies^41^^,^^42^. Furthermore, the band at 345 nm can be attributed to nAl_2_O_3_-Silane doped epoxy acrylate polymer, while the absorption band of polymer without nAl_2_O_3_-Silane is at 320 nm^43^^,^^44^. This shift is caused by the presence of scattered nAl_2_O_3_-Silane nanoparticles in the UV-cured epoxy acrylate polymer.

#### Microscopic and surface analysis

##### Scanning electron microscopy (SEM)

**Fig. 7:**
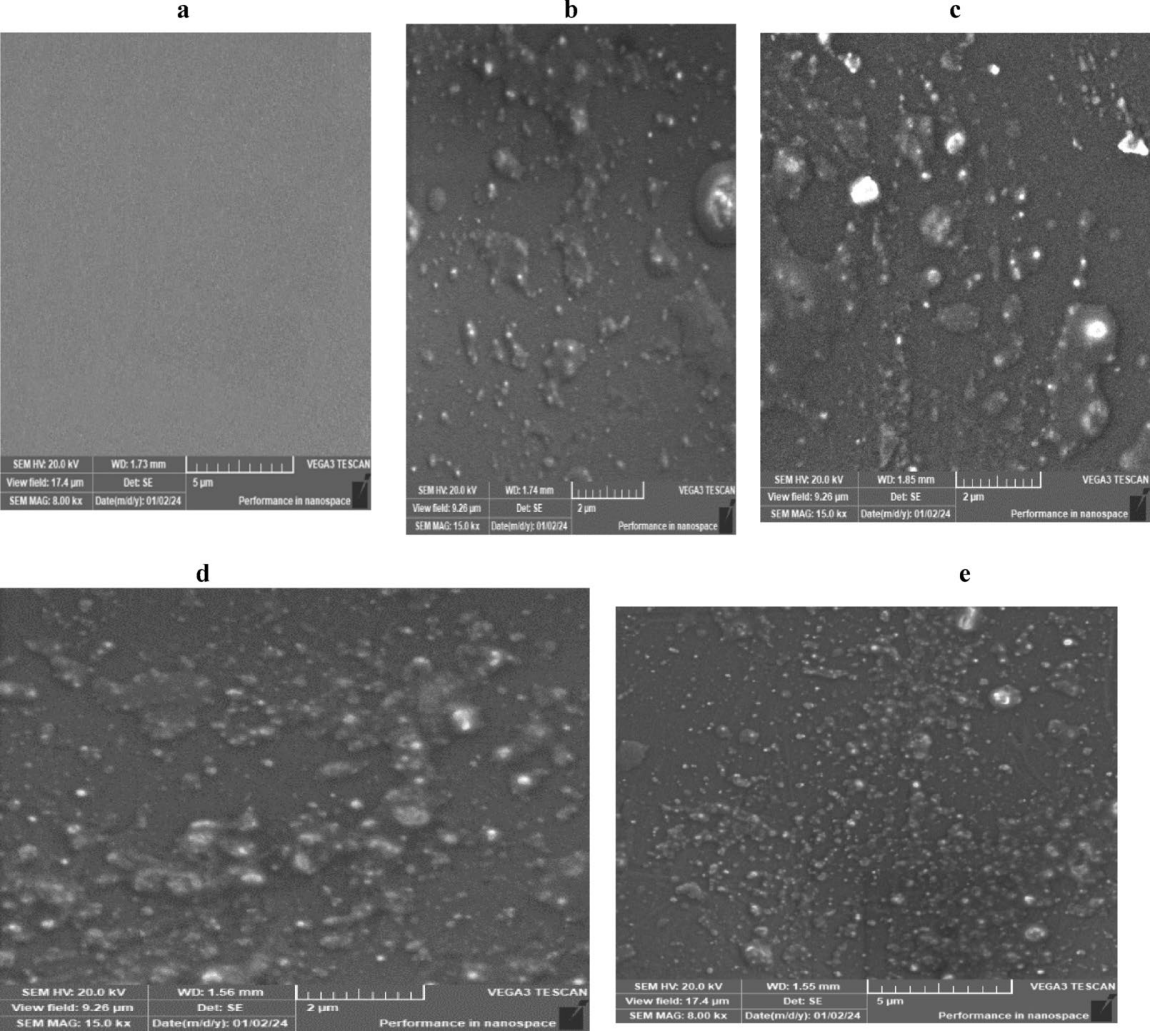
The morphological analysis of UV-cured epoxy acrylate films devoid of nAl_2_O_3_-Silane at different ratios (0% (**a**), 2% (**b**), 4% (**c**), 6% (**d**), and 8% (**e**)).

The SEM analysis of UV-cured epoxy acrylate coatings (Fig. 7) reveals the effect of varying nAl_2_O_3_-Silane concentrations on the coating morphology. The polymer without nanoparticles Fig. 7a exhibits a smooth and homogeneous surface, consistent with previous literature^45^. In contrast, the incorporation of nAl_2_O_3_-Silane nanoparticles at (2, 4, 6, and 8 wt%). Figure 7b–e introduces bright spots, corresponding to the nanoparticles, leading to increased surface roughness^46^. Additionally, SEM images confirm that nAl_2_O_3_-Silane nanoparticles are randomly dispersed within the polymer matrix.

##### Transmission electron microscopy (TEM)

**Fig. 8:**
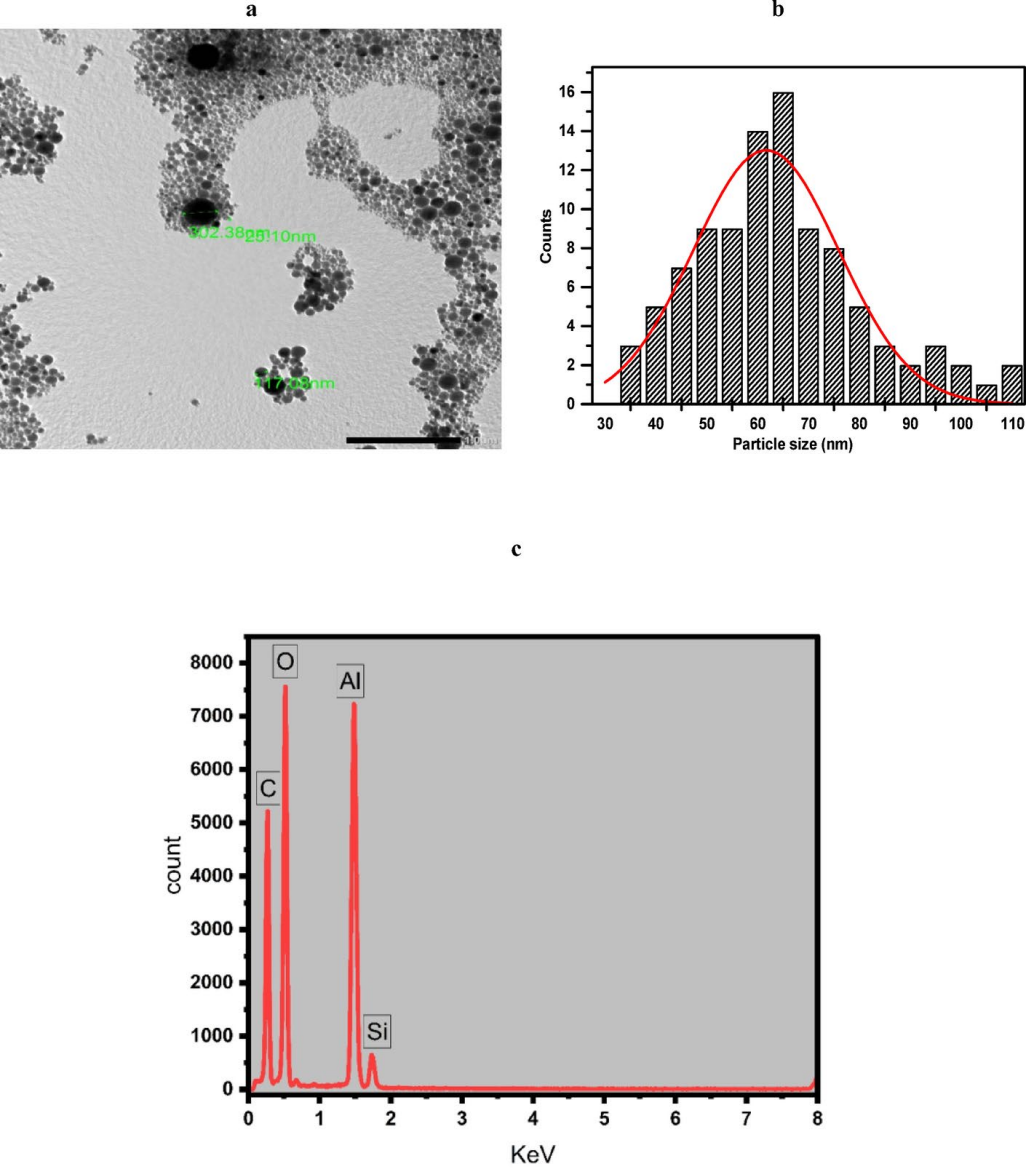
The TEM image (**a**), particle size distribution histogram (**b**), and EDX measurement (**c**) of nAl_2_O_3_-Silane dispersion.

Figure 8a presents the TEM image of nAl_2_O_3_-Silane dispersion, revealing well-distributed, non-aggregated spherical nanoparticles. Figure 8b displays the corresponding size distribution histogram, indicating a mean particle size of 61.25 nm, with sizes ranging from 30 to 100 nm. A TEM equipped with an EDX system, confirmed the presence, purity, and elemental composition of nAl_2_O_3_-Silane nanoparticles (Fig. 8c). The EDX showed characteristic peaks for carbon (C), oxygen (O), silicon (Si), and aluminum (Al) at 0.273, 0.52, 1.48 and 1.826 eV which confirms the presence of nAl_2_O_3_-Silane nanoparticles and the successful surface functionalization of alumina.

**Fig. 9:**
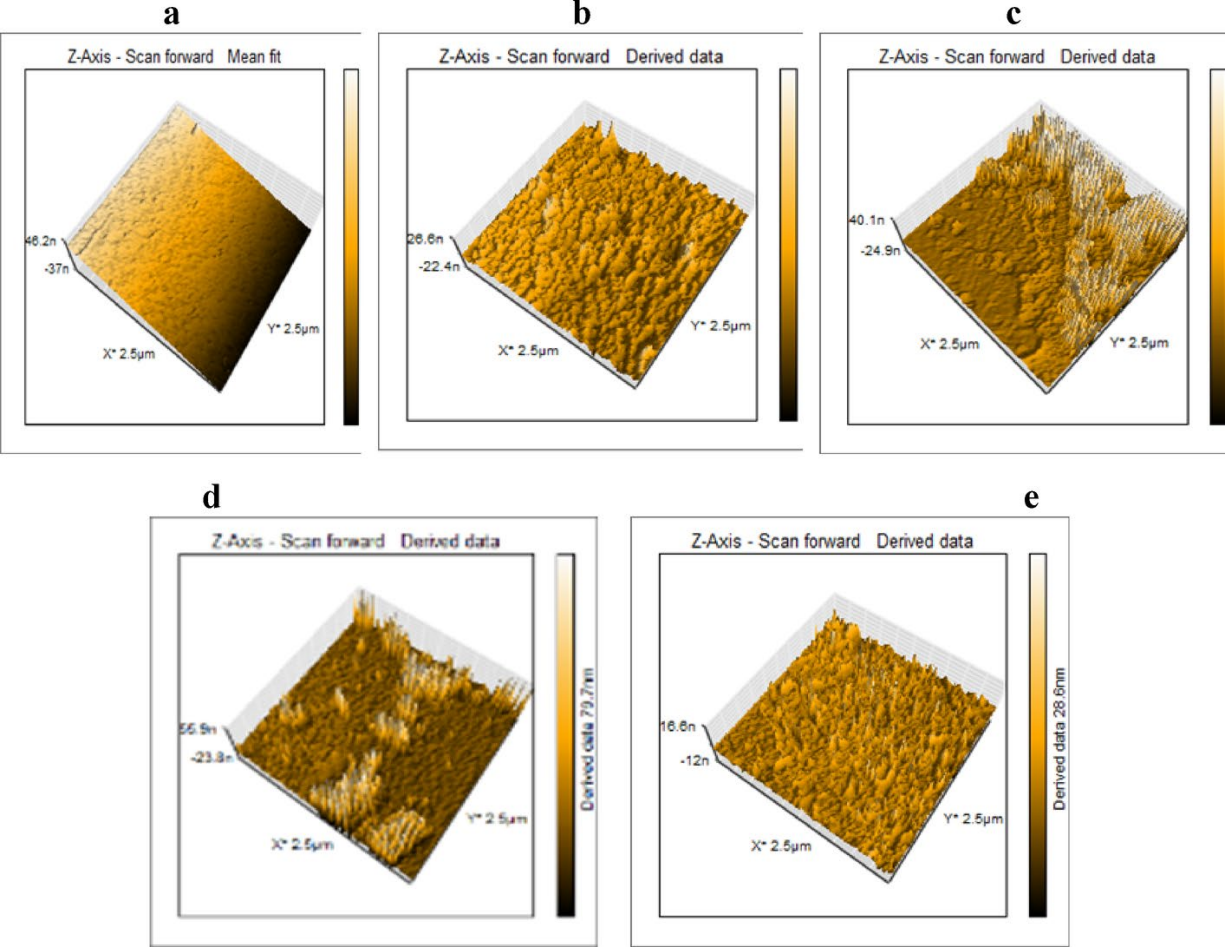
AFM images of UV-polymer surfaces prepared on a steel substrate at different concentrations (0% (**a**), 2% (**b**), 4% (**c**), 6% (**d**), and 8% (**e**)) of nAl_2_O_3_-Silane.

*Atomic force microscopy (AFM)*: AFM analysis investigated the surface topography of UV-polymer coatings with varying concentrations of nAl_2_O_3_-Silane, as shown in Fig. 9a–e. The polymer surface without nAl_2_O_3_-Silane (Fig. 9a) displayed minimal roughness (0.8 nm) and an almost flat topography. Introducing nAl_2_O_3_-Silane increased surface roughness, with *R*_a_ values rising progressively to 3.00 nm, 13.23 nm, 18.55 nm, and 21.26 nm for (2, 4, 6, and 8 wt%) concentrations, respectively. The observed roughness increase is attributed to nAl_2_O_3_-Silane aggregation, underscoring its influence on surface characteristics and coating performance^47^. These results confirm that incorporating nAl_2_O_3_-Silane into the epoxy polymer leads to an augmentation in the roughness of steel surfaces. This insight into surface characteristics is essential for understanding the impact of nAl_2_O_3_-Silane on coating properties and can guide further improvements in coating formulations^48^. The increased surface roughness enhances mechanical interlocking between the coating and the substrate, thereby improving adhesion and durability. Furthermore, the larger surface area resulting from increased roughness can enhance the coating’s corrosion resistance and minimize fouling adhesion on the surface^49^.

#### Structural analysis

##### X-ray diffraction (XRD)

**Fig. 10:**
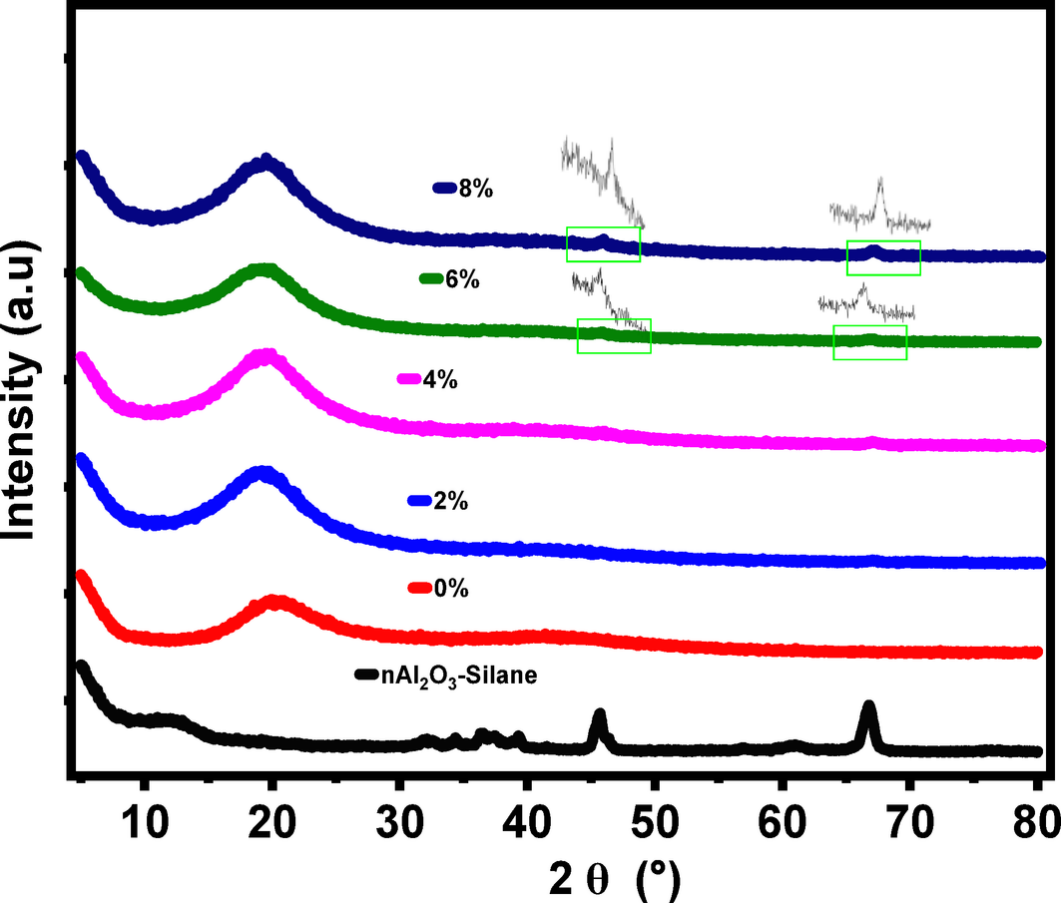
XRD pattern of the UV-polymer at different concentrations (0%, 2%, 4%, 6%, and 8 wt%) of nAl_2_O_3_-Silane.

X-ray diffraction (XRD) analysis was conducted to study the impact of incorporating (nAl_2_O_3_-Silane) nanoparticles into a UV-cured polymer matrix in a hybrid coating as shown in Fig. 10. The XRD profile of nAl_2_O_3_-Silane showed strong and intense peaks at 2θ values of 37.526°, 45.982°, and 67.184°, indicating a modified alumina structure^50^. In contrast, the epoxy acrylate polymer without the prepared nAl_2_O_3_-Silane nanoparticles appeared amorphous, with only a broad characteristic peak at 2θ = 20°^51^. It is also seen from Fig. 10 that the XRD curves of the epoxy acrylate polymers containing the (nAl_2_O_3_-Silane) nanoparticles are still amorphous despite the presence of treated alumina nanoparticles. This pattern persisted even up to (6 wt%) concentration of nAl_2_O_3_-Silane nanoparticles^52^. It is noticeable that at higher concentrations of nAl_2_O_3_-Silane nanoparticles (6 and 8 wt%), low-intensity peaks appear at 2θ = 45.8° and 67.1°, corresponding to the (nAl_2_O_3_-Silane) nanoparticles. The appearance of these low-intensity peaks can be attributed to the enhanced interaction between the polymer and the nanoparticles treated with the silane coupling agent^53^^,^^54^. This slight crystallinity especially at higher concentrations (6 and 8 wt%), does not significantly alter the inherent properties of the epoxy acrylate polymer. However, the presence of both amorphous and crystalline phases in the hybrid coating contributes to enhanced hardness and barrier properties, improving durability and environmental resistance. This, in turn, enhances mechanical performance and reduces crack propagation, which is crucial for corrosion resistance^55^.

#### Mechanical and physical properties

##### Refractive index

This study explores the use of treated nano-alumina dispersion with a reduced refractive index (~ 1.5) to enhance transparency in UV-cured coatings. The particle size critically influences light scattering and haze formation, as scattering intensity depends on the refractive index contrast between the dispersed particles and the polymer matrix (1.48). Natural aluminum oxide, with a higher refractive index (1.72), increases scattering. To minimize haze, both particle size and concentration must be controlled. The nano-alumina treated dispersion significantly reduces light scattering, improving transparency, particularly when particle size is (~ 60 nm), where Rayleigh scattering dominates^56,57^. UV-polymer coatings were applied on glass panels, and haze measurements were conducted to assess the effect of treated nano-alumina dispersion on coating clarity. As shown in Table 3, haze remained below (2%) at loading levels up to (8 wt%). The treated nano-alumina dispersion did not affect film clarity, hence minimizing haze.

##### Gel fraction

**Fig. 11:**
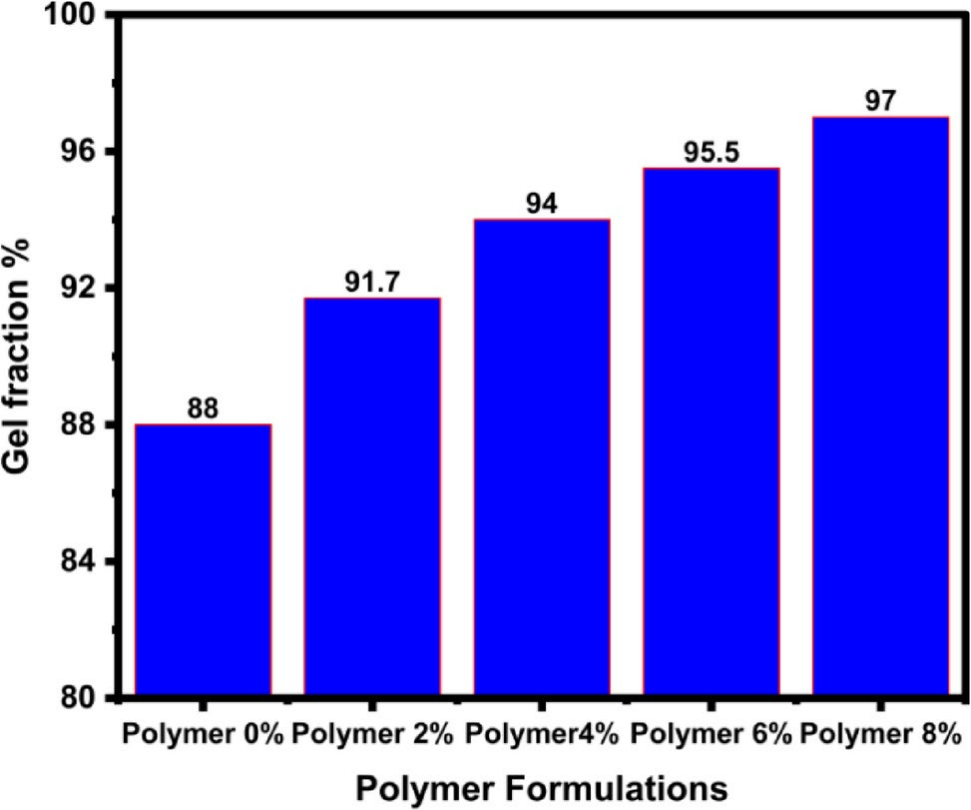
Gel fraction percentage of polymer with and without composite.

The gel fraction analysis of the cured epoxy acrylate coatings with and without the prepared nAl_2_O_3_-Silane nanoparticles is presented in Fig. 11. The gel fraction of polymers containing nAl_2_O_3_-Silane exhibits a notable increase as the nanoparticle’s percentage rises. This behavior indicates the composite’s significant impact on the gel content. The observed differences in gel content can be attributed to the contributions of functional groups and monomers on the alumina surface, influencing the polymerization conversion process^58^. A gel fraction corresponding to a crosslinking degree within the range of (88–97%) is achieved through UV curing. This is attributed to the rapid reaction of C=C double bonds in the epoxy acrylate oligomer groups and trimethylol propane triacrylate (TMPTA) with each other under UV irradiation, leading to the formation of an insoluble crosslinked structure^12^. While the quantity of epoxy groups does not play a substantial role in forming the crosslinked structure, the amount of the multifunctional component, TMPTA in this case, has a discernible impact on the degree of crosslinking. This observation suggests that the concentration of TMPTA influences the crosslinking efficiency of the cured coating^59^. The increase in gel fraction results in a higher crosslinking density, which enhances the mechanical strength, chemical resistance, and durability of the coatings. Furthermore, the dense crosslinked structure reduces permeability, thereby improving the barrier properties against corrosive agents^60^.

**Fig. 12:**
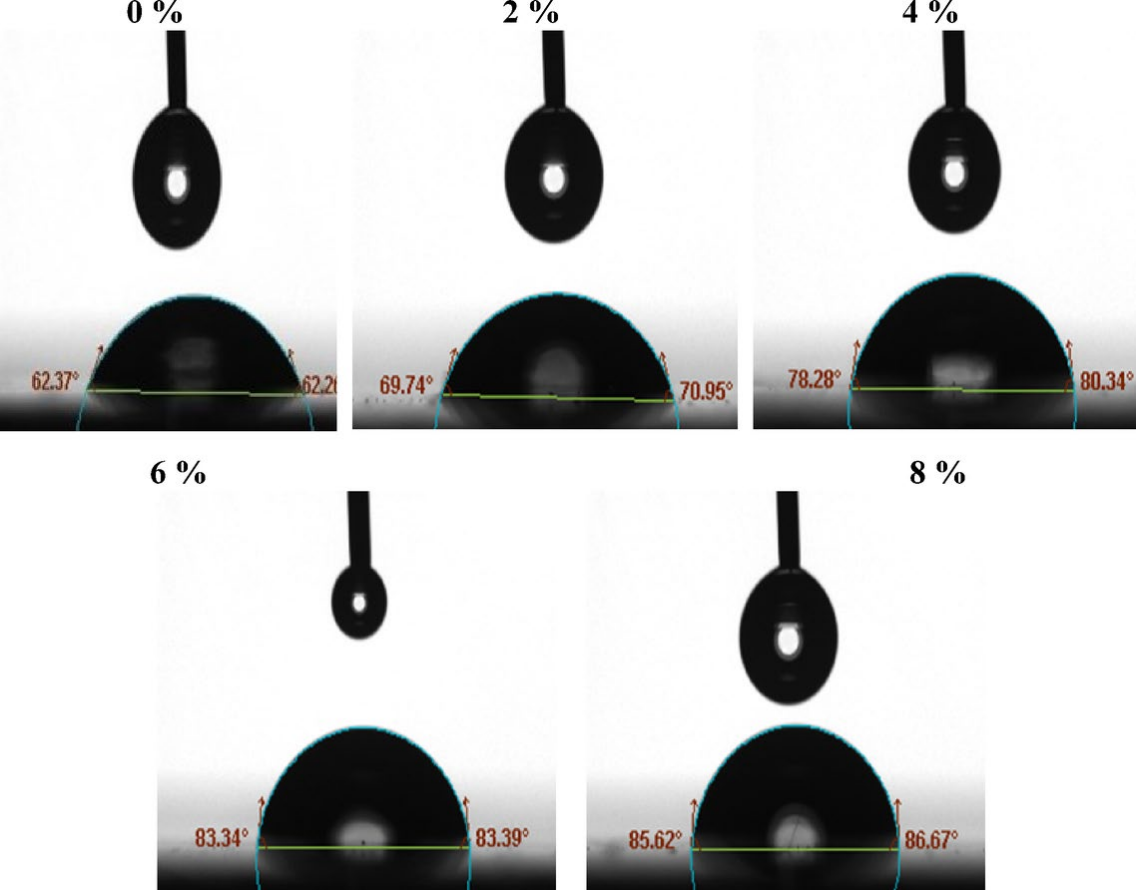
Photographs of a water droplet taken on UV-cured coating films containing nAl_2_O_3_-Silane at various concentrations.

*Contact angle*: The effects of nAl_2_O_3_-Silane concentration on epoxy coatings’ wettability and surface morphology were investigated (Fig. 12). The contact angle of epoxy acrylate UV-cured coating on a steel surface without nanoparticles was found to be 62.3°. By adding (2 wt%) nAl_2_O_3_-Silane into the polymer matrix, the liquid repellency of the surfaces was poor, and the contact angle was only 69.7°. Nanoparticle concentration is the key parameter to achieve hydrophobicity. The contact angle increased significantly with the increase of nAl_2_O_3_-Silane concentration on all of the surfaces, indicating an increase in the hydrophobicity of coating^61^, WCA values increased from 69.7° to 78.2° and 83.3° as seen in formulations of polymer with nanoparticles percentages (2, 4 and 6 wt%), respectively; the good hydrophobic surface was obtained with an angle of 86.6° at (8 wt%) of nAl_2_O_3_-Silane nanoparticles, this is due to the excellent diffusion of nAl_2_O_3_-Silane in the polymer matrix, which resulted in a hydrophobic surface with increasing WCA values. and the addition of nanoparticles could change the micro/ nanostructure of the coating. The surfaces perform good hydrophobicity properties at the higher nanoparticle contents^62^. These results reported the importance of well-distributed nanoparticles in the UV-polymer coating nanocomposites for coating’s corrosion protection application. Good interactions between the polymer matrix and the prepared nanoparticles resulted in increased adhesion and flexibility of the coating to the substrate. The formation of a cross-linked network and strong interfacial interaction between well-dispersed nanoparticles and the epoxy surface could be interpreted as a significant determinant for surface durability. Chemisorption and/or physisorption eventually led to the formation of physical networks.

*Solvent and chemical resistance*: The incorporation of nAl_2_O_3_-Silane nanoparticles into the UV-polymer matrix significantly enhanced its solvent and chemical resistance. This improvement can be due to the polymer network’s enhanced crosslinking density, as demonstrated by the gel fraction test, which reduces solvent permeability and aggressive species. The MEK rub test confirmed the high solvent resistance, as the composite coatings exhibited no significant degradation. In terms of chemical resistance, the weight loss measurements after immersion in (10%) HCl and (10%) NaOH indicated minimal degradation, with no substantial changes in appearance except for slight yellowing in NaOH exposed samples and the results are tabulated in Table 2. This can be explained by the improved structural integrity of the coating due to the presence of nAl_2_O_3_-Silane, which acts as a reinforcing agent, reducing polymer chain mobility and limiting chemical penetration. Additionally, the silane treatment enhances adhesion between the nanoparticles and polymer matrix, further contributing to the overall durability and resistance of the coatings^63,64^.

**Table 2:** Degree of chemical resistance of UV-cured polymer nanocomposites.

Polymer (wt%) filler	MEK rub resistance cycles	(10%) HCl		(10%) NaOH	
		Appearance	Weight loss (%)	Appearance	Weight loss (%)
0	82	Good	2.5%	Yellowish	4%
2	90	Good	> 1%	Slight yellowish	2.5%
4	95	Good	> 1%	Good	> 1%
6	100	Good	> 1%	Good	> 1%
8	100	Good	> 1%	Good	> 1%

*Scratch test*: The scratch resistance results for samples, both with and without nAl_2_O_3_-Silane nanoparticles, are comprehensively presented in Table 3. It is well-established that the incorporation of reinforcing nanoparticles generally enhances the mechanical properties of polymers^65^. nAl_2_O_3_-Silane nanoparticles introduced into the polymer matrix to augment their mechanical properties. The polymer samples containing the nAl_2_O_3_-Silane nanoparticles exhibited robust bond strength, attributed to mechanical interlocking within the transfer coating on the counter face asperities, without involving any chemical interaction. Hard inorganic particles within the polymer matrix effectively divided the single lateral force into different force components. Consequently, the force required to induce a scratch in the UV-curable coating containing nAl_2_O_3_-Silane nanoparticles was notably higher compared to those without nAl_2_O_3_-Silane nanoparticles^66^, the scratch resistance of the polymer coating nanocomposites was investigated with the nanoparticles content. The UV-polymer coatings without nanoparticles exhibited a scratch resistance of approximately 14.7 N. With the addition of (2, 4, 6, and 8 wt%) of nAl_2_O_3_-Silane nanoparticles, the scratch resistance progressively increased to 19.6 N, 29.4 N, 34.3 N, and 39.2 N, respectively. The highest scratch resistance was observed in coatings with (6 wt%) and (8 wt%) of nAl_2_O_3_-Silane, demonstrating a (131%) improvement over the polymer coating without composite^67^. Similar studies have reported enhanced scratch resistance in polymer coatings due to the incorporation of inorganic nanoparticles, which improve mechanical durability by reducing plastic deformation and wear^68,69^. Furthermore, the addition of silane-functionalized nanoparticles has been shown to improve interfacial adhesion within polymer networks, further contributing to enhanced mechanical properties^58,70^. These findings align with previous research demonstrating that nanoparticle-reinforced coatings exhibit superior tribological properties due to their ability to reduce friction and increase resistance to surface damage. The scratch test results revealed increased resistance, attributed to the elevated surface roughness of the coatings with higher concentrations of nAl_2_O_3_-silane nanoparticles, corroborating the AFM findings.

**Table 3:** Mechanical and physical properties of UV-cured polymer nanocomposites.

Polymer (wt%) nAl_2_O_3_-Silane	Gloss 60^o^		Haze (%)	Adhesion	Scratch (N)	
	Value	Stander deviations			Value	Stander deviations
0	108°	1.04°	0	3B	14.7	0.82
2	106°	1.32°	0.27	4B	19.6	0.49
4	102°	1.53°	0.61	4B	29.4	1.39
6	97°	0.76°	1.05	5B	34.3	0.49
8	95°	1.01°	1.48	5B	39.2	1.61

*Gloss measurement*: The gloss observed in the painted panels can be ascribed to surface characteristics, including smoothness^71^. The gloss readings of the surface coatings reveal that the UV-cured epoxy acrylate/nAl_2_O_3_-Silane coatings exhibit high gloss. Table 3 provides insights into the glossiness of UV-epoxy acrylate coatings with varied compositions of nAl_2_O_3_-Silane. The baseline gloss for neat epoxy acrylate coatings was measured at 108 GU. Including up to (4 wt%) of nAl_2_O_3_-Silane dispersion does not impact the glossiness of the coatings. However, an increase in the nAl_2_O_3_-Silane content to (8 wt%) reduces glossiness to 95 GU. This decrease can be attributed to lower quantities of nAl_2_O_3_-Silane, enabling smoother and more uniform coating flow. Additionally, smaller nanoparticles with a spherical appearance and a narrow size distribution influence the high glossiness value. These particles fill surface irregularities, yielding a smoother surface with more compact air boundary surfaces^72–74^. Moreover, the polymer coating film, enriched with reactive nAl_2_O_3_-Silane, produces films characterized by low turbidity and high optical clarity, even at loading levels as high as (8 wt%) Previous studies have shown that the loading of nanoparticles generally leads to a significant decrease in gloss^40^. However, in our study, the reduction in gloss was not as pronounced due to the uniform structure of nAl_2_O_3_-Silane and the absence of surface defects after its incorporation. Compared to these systems, the UV-cured epoxy acrylate/nAl_2_O_3_-Silane coatings offer a unique balance of high gloss, smooth surface morphology, and optical clarity, making them particularly advantageous for applications requiring.

### Cross cut adhesion

The cross-cut tape adhesion test was conducted to evaluate the adhesion strength of the epoxy acrylate coating on a steel substrate. The adhesion strengths of all UV-polymer coating composites are presented in Table 3. The UV-polymer coating without prepared nanoparticles (0 wt%) exhibited an adhesion strength of 3B, which is comparatively lower than that of UV-polymer coatings containing nanoparticles. Specifically, coatings incorporating n-Al_2_O_3_-silane nanoparticles demonstrated adhesion strengths in the range of 4B to 5B. This result surpasses the adhesion strength of polyurethane derived from soybean oil-based polyol^75^, and aligns with the performance of UV-curable polyurethane acrylate/Al_2_O_3_ nanocomposite coatings^67^. In general, the adhesion strength of ESOA@TMPTA-nAl_2_O_3_-silane coatings increased with nanoparticles loadings up to (8 wt%) nAl_2_O_3_-Silane. It is well established that adhesion is influenced by mechanical interlocking and surface roughness^76^. Increasing the nanoparticle content in the UV-polymer matrix enhances surface roughness (as confirmed by AFM analysis), promoting mechanical interlocking and adhesion strength. This improved adhesion delays electrolyte penetration, thereby enhancing corrosion resistance^15^. Moreover, the high crosslinking density, as indicated by gel fraction results, further strengthens adhesion to the metal substrate^77^.

#### Corrosion resistance testing

##### Corrosion measurements

**Fig. 13:**
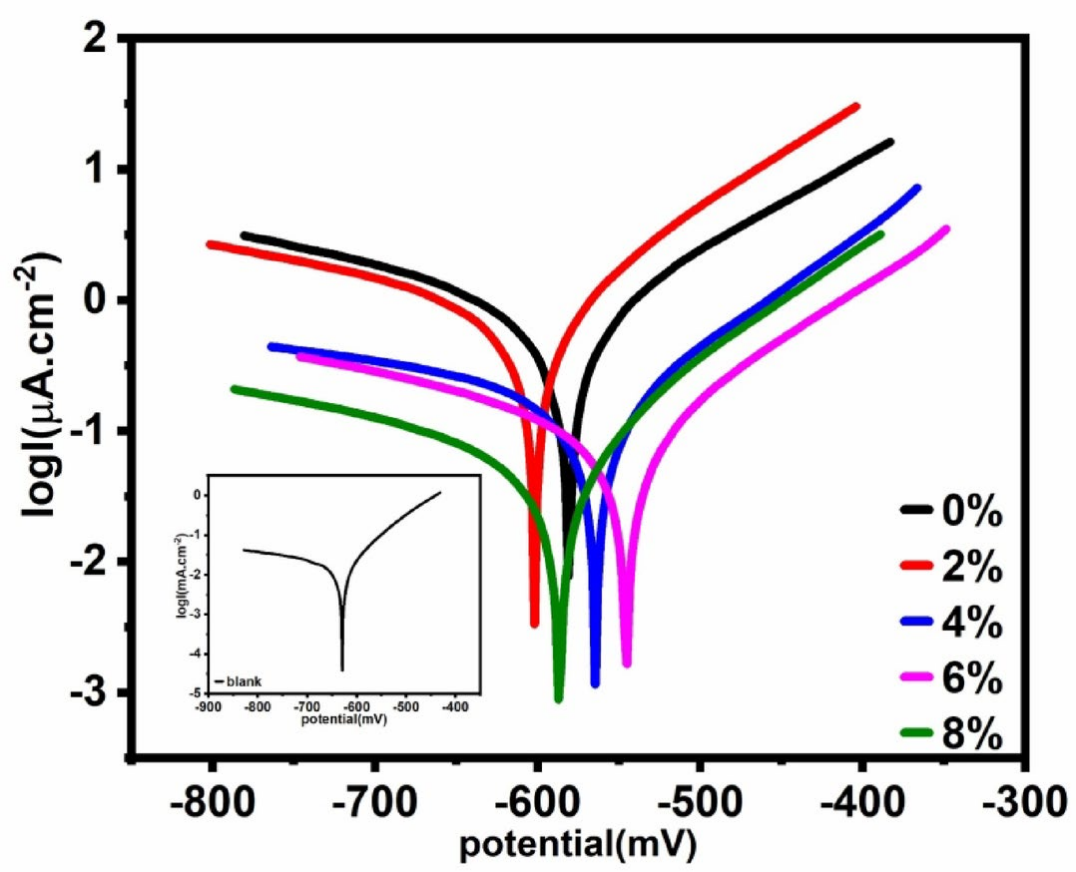
Potentiodynamic polarization curves in (3.5 wt%) aqueous NaCl for carbon steel painted with different formulations.

*Potentiodynamic polarization measurement*: The cathodic and anodic polarization techniques of metal are frequently used to study the phenomena of metal corrosion and passivation^78^. Tafel polarization plots were recorded for carbon steel electrode coated with UV-cured epoxy acrylate coating with and without nAl_2_O_3_-Silane in (3.5 wt%) NaCl solution at 30 °C were represented in Fig. 13. The polarization response of the electrode coated with a UV-curable coating containing different concentrations (2, 4, 6, and 8 wt%) of nAl_2_O_3_-Silane is shifted toward both anodic and cathodic sides. This indicates that the nAl_2_O_3_-Silane may inhibit both the anodic and cathodic reactions and enhance the corrosion resistance of the coating. The value of *i*_corr_ for electrodes coated with epoxy acrylate polymer (8 wt%) nAl_2_O_3_-Silane is higher than those for the electrodes coated with UV-curable epoxy acrylate containing various amounts (0, 2, 4, 6 wt%) of nAl_2_O_3_-Silane as shown in Table 4. The results reveal that nanocomposite’s presence improves the corrosion protective power of the UV-cured epoxy acrylate polymer. The presence of nanoparticles blocks the pores and holes of the polymer coating^79^. The dispersion of nAl_2_O_3_-Silane can penetrate, disperse quickly, and integrate into the polymer structure to cover the micropores and decrease permeability^80^^,^^81^, the inhibition efficiency () was calculated from the polarization data as follows^82^, as shown in Eq. (2).2where ***i⁰***_**corr**_ and ***i***_***c*orr**_ are the corrosion current densities for steel with and without polymer coatings, respectively.

**Table 4:** Data obtained from polarization measurements characterizing the corrosion behavior of the painted steel in (3.5 wt%) NaCl solution.

(%)	CR (μm/Y)	*i*_corr_ (_µA/cm_^2^)		*ß*_a_ _(mV dec_^-1^_)_	*-ß*_c_ _(mV dec_^-1^_)_	*-E*_*corr*_ _(mV vs. SCE)_	Conc.^a^ (wt%)
		*Value*	*Standard deviation* × *10*^*–2*^				
–	218.62	18.7	62.64982	103.5	244.2	629.0	Blank
95.57	9.67	0.827	3.513308	154.0	280.3	581.2	0
96.37	7.9	0.678	1.001665	115.1	169.7	601.8	2
98.95	2.28	0.195	0.264575	133.2	552.8	564.2	4
99.63	0.81	0.069	0.2	117.2	390	545.3	6
99.68	0.68	0.059	0.152753	113.0	340.9	586.7	8

a= Conc. of nAl_2_O_3_-Silane in polymer matrix

The data in Table 4 show that the inhibition efficiency increases with increasing nanoparticle concentration. The inhibition efficiency of the paints investigated increased in the following order: 0 < 2 < 4 < 6 < 8. The obtained polarization data showed that the sample with nAl_2_O_3_-Silane nanoparticles presented a better barrier effect against electrolyte penetration than in its absence. This effect increases with the added number of additives.

Data in Table 4 showed that following the incorporation of the nAl_2_O_3_-Silane nanoparticles, the variation of *E*_corr_ indicates that the nanoparticles operate as a mixed-type inhibitor. Additionally, the slopes (*β*_*a*_) and (*β*_*c*_) of the anodic and cathodic Tafel lines are almost independent of paint composition, which means that changing the amount of nAl_2_O_3_-Silane in the coating does not significantly change the mechanism of the corrosion process. So, modification’s role is mainly in blocking the active reaction sites inside the pores and holes. When the coated specimens were exposed to (3.5 wt%) NaCl solutions, the medium’s corrosive agents (H_2_O, Clˉ ions) reached the metal/coating interface through the pores and corroded the base metal surface (active sites). Rust undercoating probably possesses a high adherence to the metal surface^83^.

Despite the high content of nAl_2_O_3_-Silane in the coating (8 wt%), no apparent agglomeration occurs due to good dispersion. Consequently, the modified coatings exhibit significantly improved barrier properties against corrosive agents such as chloride ions and water. Consequently, the corrosion resistance of the modified coating is very high. In some research^84^, the coating becomes porous with a high load of fillers in organic coating system beyond composite value. The filler can easily agglomerate and is converted to micron size because of a large specific surface area and high surface activity^85^. The addition of nAl_2_O_3_-Silane nanoparticles shifted the Tafel curves toward more noble (passive) regions by reducing both anodic and cathodic current densities. This effect is attributed to the blocking of both cathodic and anodic sites. The hydrogen reduction reaction was controlled by activation energy, which can be explained by the obstruction of corrosion-active sites on the carbon steel surface. This blockage reduces the available surface area for anodic reaction. In the anodic region, the anodic Tafel lines indicate the protective ability of nAl_2_O_3_-Silane nanoparticles in shielding carbon steel from aggressive environments. As shown in Fig. 13, the presence of nAl_2_O_3_-Silane nanoparticles inhibits both anodic carbon steel dissolution and cathodic hydrogen evolution. This behavior is associated with an increase in the concentration of nAl_2_O_3_-Silane nanoparticles^86^. Based on the *E*_corr_ values and the anodic and cathodic Tafel slopes, no modification in the corrosion reaction mechanism of carbon steel was observed^87^.

**Fig. 14:**
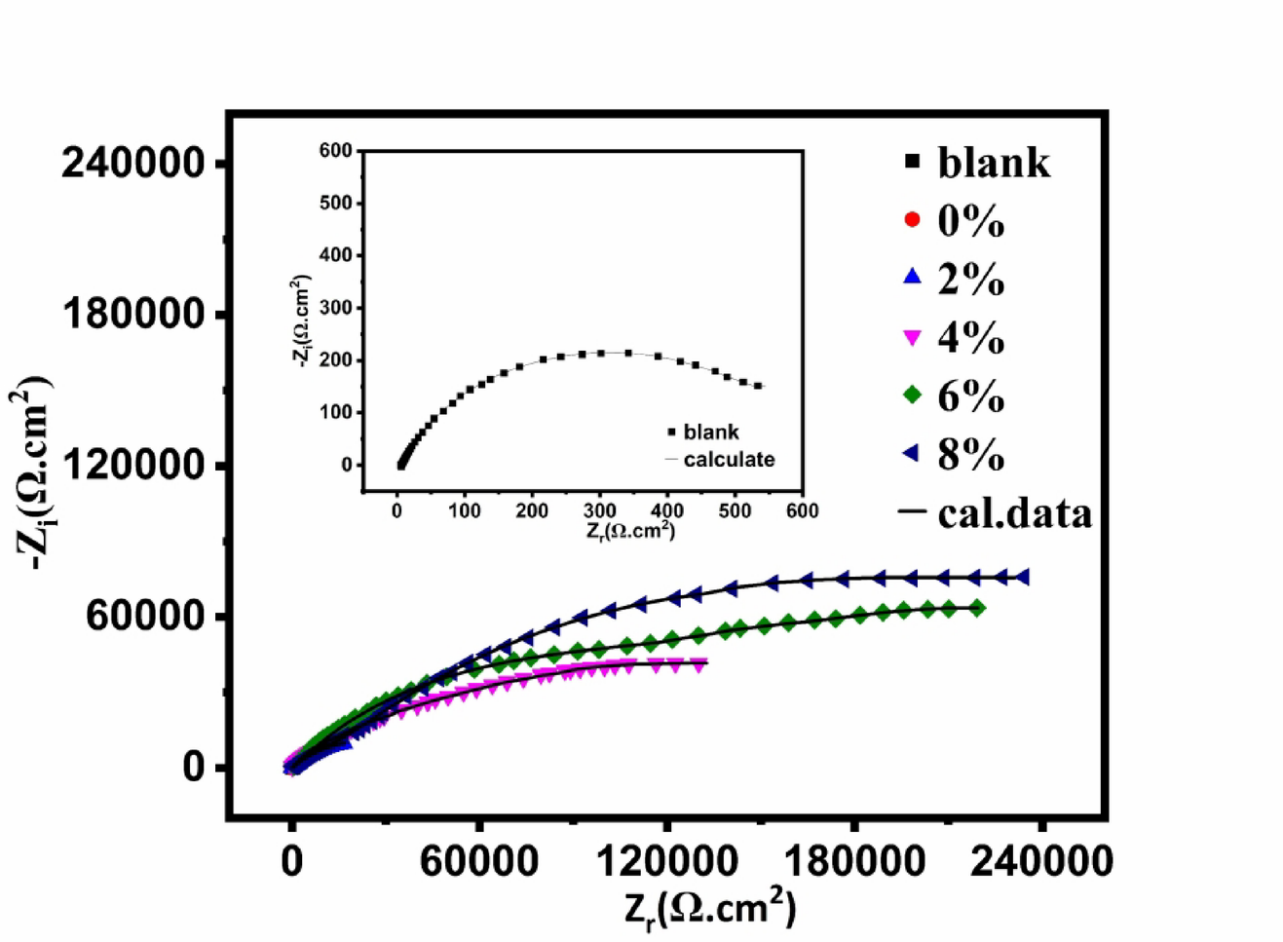
Nyquist plot of coating systems for carbon steel painted with the different paint formulations after immersion in (3.5 wt%) aqueous NaCl.

**Fig. 15:**
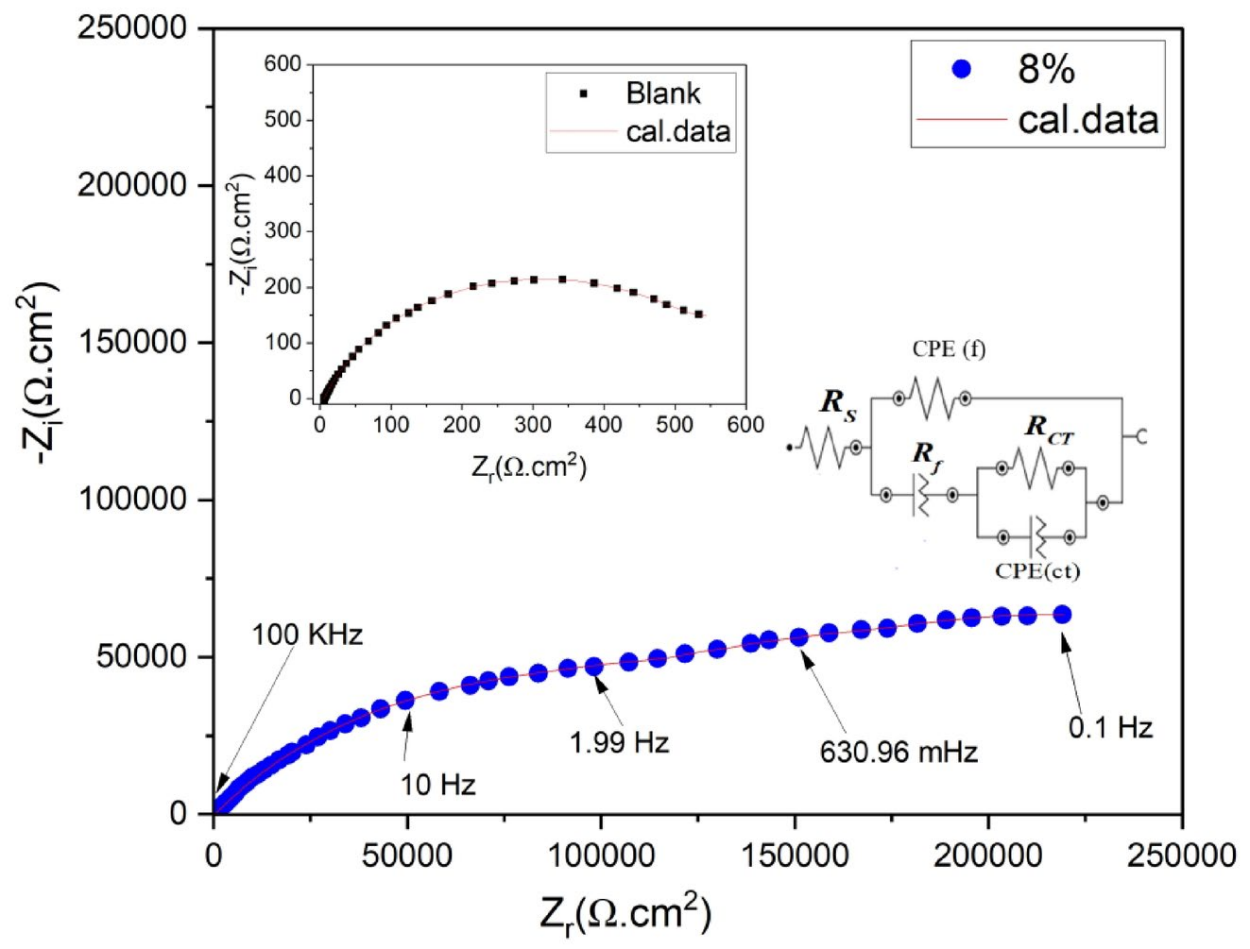
Nyquist plot of carbon steel in NaCl solution with and without UV-polymer (8 wt%) n-Al_2_O_3_-Silane using the proposed equivalent circuit.

**Fig. 16:**
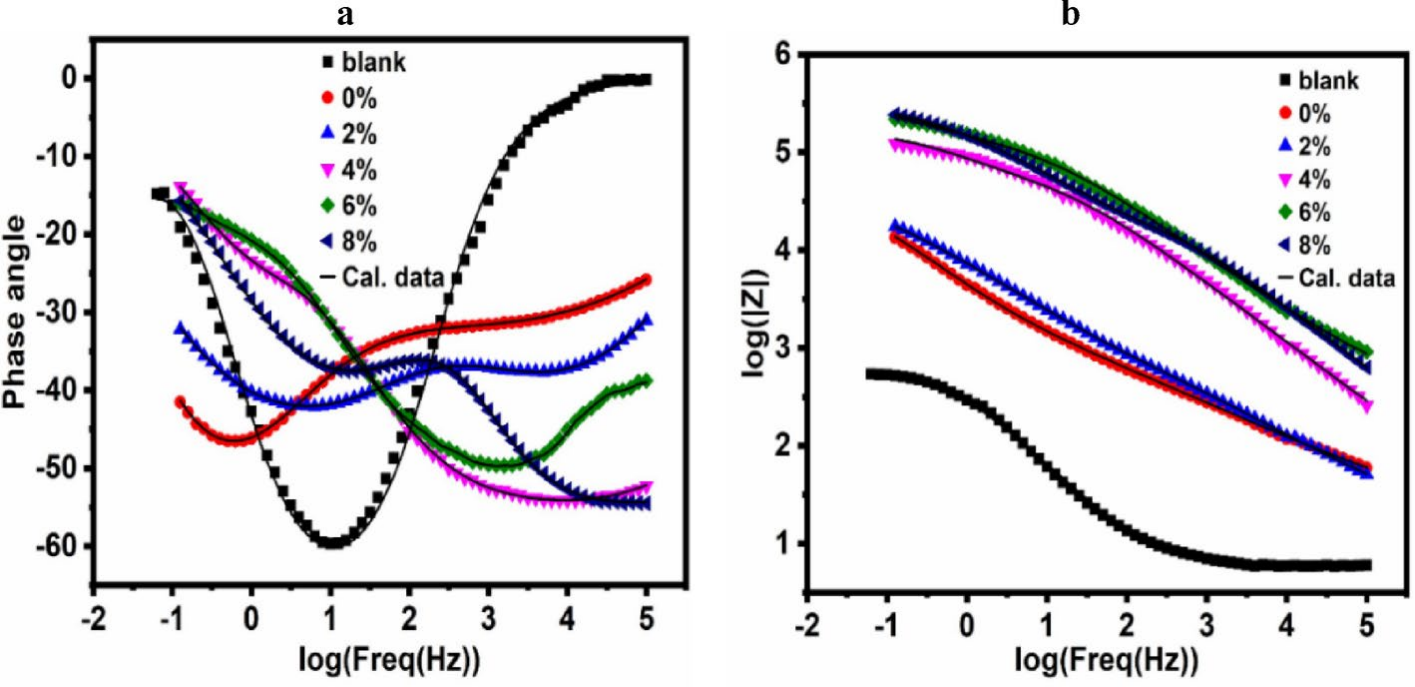
Bode plot for carbon steel painted with the different paint formulations after immersion in (3.5 wt%) solution NaCl.

*Electrochemical impedance spectroscopy (EIS)*: Electrochemical impedance spectroscopic measurements were recorded for carbon steel electrode coated with UV-cured epoxy acrylate coating with and without nAl_2_O_3_-Silane in (3.5 wt%) NaCl solution at 30 °C and shown in Fig. 14. The Nyquist plots (real impedance vs imaginary impedance). The UV-polymer coatings showed a non-perfect semi-circle on the Nyquist plot, the curve’s diameter shows the polarization resistance (*R*_p_); a greater *R*_p_ value denotes improved corrosion protection^88^. The corrosion protection of a coating can be estimated using *R*_p_. Because of the increased barrier layer thickness, the Nyquist plot’s curve diameter grows when the nanoparticles percentage rises to (8 wt%). At modest loading of nAl_2_O_3_-Silane (2, 4, 6, and 8 wt%) particles, the resistance of nanocomposite coatings against electrolyte diffusion is enhanced. Because the hydrophobic nature of nanoparticles delayed surface wetting and prevented corrosive ions from passing through the coating layer, they effectively created a barrier^89^. The chemical interaction between functional groups on polymer and nanoparticles can enhance the barrier property and ionic resistance of UV-polymer coating composites^20^. Compared to other nAl_2_O_3_-Silane content percentages of coating, the capacitive arc diameter for samples containing (8 wt%) nAl_2_O_3_-Silane was higher. This further demonstrates how nanoparticles can have a locking effect on metal surfaces, preventing electrolytes from reaching the coating-metal contact. The impedance diagram in the presence of nAl_2_O_3_-Silane nanoparticles at high frequencies exhibits a capacitive loop, which can be attributed to the double-layer capacitance, indicating that the mitigation process is governed by charge transfer. At low frequencies, an incomplete capacitive loop is observed, suggesting the formation of an nAl_2_O_3_-Silane coating layer that protects the metal surface from the surrounding environment. These observations indicate that the Nyquist curves consist of two-time constants, a finding further confirmed by the Bode-phase diagram, as shown in Fig. 16. The proposed equivalent circuit (EC), illustrated in Fig. 15, can be represented as follows: *R*_s_ (solution resistance), *R*_p_ = *R*_ct_ (charge transfer resistance) + *R*_f_ (film resistance), and *CPE* (constant phase element), which is characterized by *Y*^o^ and the coefficient *n*. Table 5 presents the protection efficiency (*η*) of the nanocomposite coatings along with the impedance characteristics obtained from the equivalent circuit. The protection efficiency (*η*) is calculated using the following Eq. (3):3where *R*_p_ and *R*^*o*^_p_ are the polarization resistance of carbon steel with and without polymer coatings, respectively.

**Table 5:** EIS parameters for painted steel at different concentrations of the synthesized nanocomposite in 3.5% NaCl solution.

Conc.^a^ (wt%)	*R*_S_ (Ω cm^2^)	C_dl_ (µF cm^2^) 10^6^	*R*_ct_ (kΩ cm^2^)	CPE_ct_		*R*_*f*_ (kΩ cm^2^)	CPE_f_		*R*_p_ (kΩ cm^2^)		(%)
				Y_0_ (μΩs^n^cm^-2^) 10^–5^	n		Y_0_ (μΩs^n^cm^-2^) 10^–7^	n	value	*Standard deviation* × *10*^*–2*^	
Blank	10.15	447.00	0.428	59.72	0.78	0.198			0.626	1.050397	–
0	10.53	123.11	0.532	7.458	0.68	25.132	1325	0.38	25.664	17.40412	98.7
2	12.78	18.22	0.629	1.872	0.80	33.105	2.923	0.80	33.734	63.49622	98.9
4	11.65	2.31	61.615	0.4687	0.48	41.818	8.034	0.61	103.433	115.9023	99.5
6	13.55	1.45	152.742	0.2037	0.61	49.862	9.825	0.67	202.604	153.2155	99.8
8	14.84	0.91	234.324	0.1631	0.55	54.405	3.260	0.69	288.729	101.4889	99.9

a= Conc. of nAl_2_O_3_-Silane in polymer matrix

Consequently, the non-ideal capacitive behavior of the double layer is represented by CPE rather than *C*_dl_, the double layer capacitance. The following Eq. (4) describes its impedance.

4 $${Z}_{\text{CPE}}=\frac{1}{{Y}_{\text{o}}{\left(j\omega \right)}^{n}}$$

where ${Y}_{\text{o}}$ is a proportional factor, ${J}^{2}=-1$, $\omega =2\pi f$ and *n* is the phase shift. For $n=0$, *Z*_CPE_ represents a resistance with $R={{Y}_{\text{o}}}^{-1}$, for $n=1$ a capacitance with $C={Y}_{\text{o}}$, for $n=0.5$ and for $n=-1$ an inductive with $L={{Y}_{\text{o}}}^{-1}$.

The uniformity of the coating thickness across different samples was controlled by optimizing curing parameters such as UV source intensity, curing time, and application techniques. According to the Helmholtz model, the coating behaves like a parallel plate capacitor, where the coated film thickness (*T*) reduces the exposed metal surface area (*A*), and the double-layer capacitance (*C*_dl_) can be calculated according to the following Eq. (5):

5 $$C_{{{\text{dl}}}} = \left( {\varepsilon ^{^\circ } \varepsilon /T} \right)S$$

where *ε°* is the permittivity of air, *ε* is the local dielectric constant, and *C*_dl_ is the capacitance of the double layer^90^. The protective action of the prepared coating composites can be attributed to its ability to isolate the carbon steel surface from the corrosive medium, thereby reducing *C*_dl_^91^.

Table 5 shows that, compared to the polymer without nanoparticles (0 wt%), all nanocomposite coatings exhibit higher polarization resistance and a lower *Y*^o^ value. The increase in *R*_p_ is most pronounced in the UV-polymer coating containing (8 wt%) nAl_2_O_3_-Silane. This enhancement in *R*_p_ can be attributed to the passivation of the metal substrate due to the incorporation of the nanoparticle’s concentration material into the coating^92^. The coating capacitance reflects the electrolyte and water ions that penetrate the coating reflects the electrolyte and water ions that penetrate the coating reflects the electrolyte and water ions that penetrate the coating. The coating capacitance of the UV-polymer with nAl_2_O_3_-Silane was lower than that of the UV-polymer without nAl_2_O_3_-Silane, suggesting that the coating provides protection in a (3.5 wt%) NaCl solution and absorbs less water^93^. A passive coating formed on the metal substrate, indicating that it is well-protected from corrosion, as indicated by an increase in *R*_p_ and a decrease in coating capacitance^94^. This clarified why adding nAl_2_O_3_-Silane nanoparticles at an (8 wt%) concentration increases carbon steel corrosion resistance. Scratch, adhesion, and water contact angle test data further validate the corrosion prevention capabilities of UV-polymer coatings containing (8 wt%) nAl_2_O_3_-Silane. The gap between the Bode curves of the prepared polymer coating nanocomposites and the uncoated carbon steel, as shown in Fig. 16, increases with the rising nanoparticles concentration. The improved protection of the carbon steel surface suggests that the mitigation efficacy of nAl_2_O_3_-Silane is concentration-dependent, as evidenced by the shift of Bode curves to higher values. Furthermore, the phase curves exhibit two-time constants, reflecting the enhanced protective performance (increasing impedance) of the UV-polymer coating composites shielding carbon steel from the corrosive environment^95^.

Figure 16 displays the UV-polymer with and without nAl_2_O_3_-Silane nanoparticles Bode modulus graphs (log *f* vs. log *Z*). The impedance modulus |*Z*| for the UV-polymer (0 wt%) nAl_2_O_3_-Silane nanoparticles is at a low frequency (100 m Hz) of (3.5 wt%) NaCl solution, as indicated in the Bode data Fig. 16a,b. On the other hand, UV-polymer coating composites show a notable improvement in value. It is, therefore, possible to conclude that the inclusion of nAl_2_O_3_-Silane improved the anti-corrosion performance of nanocomposite coatings by enhancing the inhibitory effect of barrier property on UV-polymer coating composites by preventing electrolyte leakage into coating. (8 wt%) of nAl_2_O_3_-Silane coatings exhibit the most noticeable rise in impedance value. This is explained by the (8 wt%) nAl_2_O_3_-Silane ‘specimens’ unique electroactivity, which was the highest value and showed the carbon steel plate’s strongest anti-corrosion capacity in a (3.5 wt%) NaCl solution. The straight line’s "−1" slope in the frequency region demonstrated the coating’s capacitive behavior and high barrier property.

Previous studies on UV-polymer coating composites under similar conditions utilized ZnO nanoparticles up to 5% for corrosion resistance, resulting in an improved current density (*i*_corr_) of 1.3 nA/cm^2^ and a corrosion efficiency exceeding 99%, which aligns with our findings^15^.

**Fig. 17:**
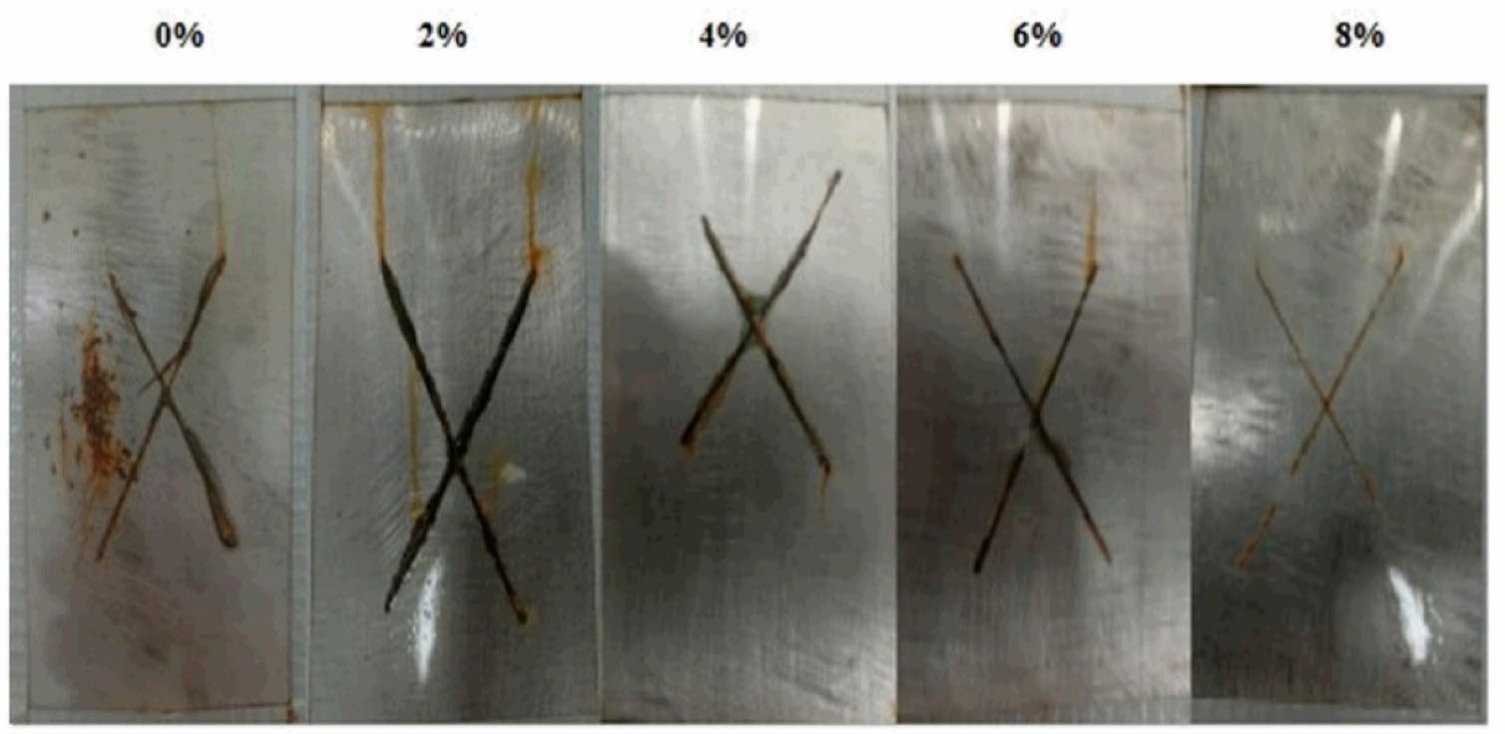
The visual analysis of the UV-polymer coating composites prepared on a steel substrate at different concentrations (0%, 2%, 4%, 6%, and 8%) of nAl_2_O_3_-Silane.

*Salt spray test*: The salt spray test was conducted to evaluate the corrosion resistance of UV-curable coatings applied to steel substrates. The assessment focused on the degree of rust formation beneath the coated films and the extent of adhesion loss at the X-scratch, following exposure to a corrosive environment. To investigate the influence of nAl_2_O_3_-Silane nanoparticles on corrosion resistance, different concentrations were incorporated into the UV-curable coating. The results of this test are visually represented in Fig. 17, highlighting the distribution of corrosion as either spot rust or general rust formation.

**Fig. 18:**
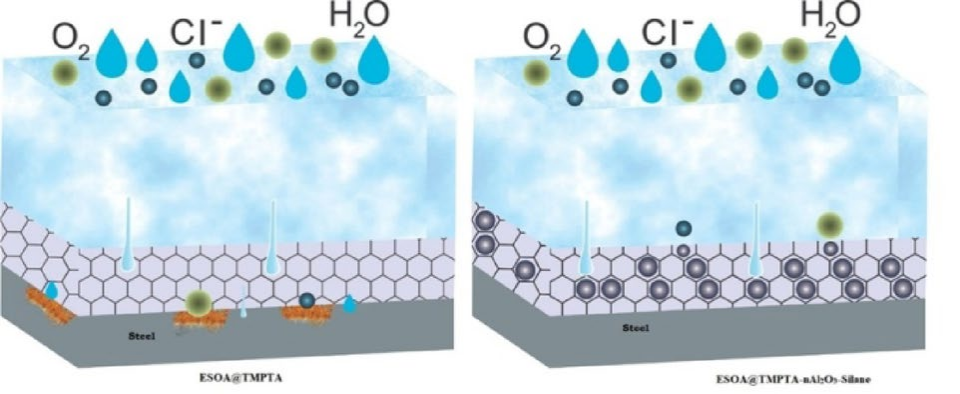
Schematic diagrams of corrosion protection mechanism for coating systems exposed to 3.5 wt% NaCl solution.

The UV-polymer coating without nAl_2_O_3_-Silane exhibited severe rust formation and significant adhesion failure around the scribe, with an affected area exceeding (10%), corresponding to a corrosion rating of (3G). However, coatings incorporating nAl_2_O_3_-Silane nanoparticles demonstrated a notable enhancement in corrosion resistance. Specifically, the UV-polymer coating containing (2 wt%) nAl_2_O_3_-Silane nanoparticles exhibited no visible rust spots (rating 5G) and only minor adhesion failure around the scribe (less than 3%). Increasing the nAl_2_O_3_-Silane nanoparticles concentration to (4 and 6 wt%) further improved corrosion resistance, achieving corrosion ratings of (6G) and (7G), respectively. The rust formation in these coatings was reduced to less than 1%, with no adhesion failure observed. Notably, the UV-polymer coating with (8 wt%) nAl_2_O_3_-Silane nanoparticles provided the highest level of protection, attaining a corrosion rating of (8G), with rust formation reduced to less than 0.1%. The enhanced corrosion resistance observed in coatings incorporating nAl_2_O_3_-Silane nanoparticles can be attributed to the formation of a robust physical barrier^27^, as illustrated in Fig. 18 which effectively blocks corrosive species from penetrating the coating. UV-polymer coatings nanocomposites act as a physical and chemical barrier, reducing direct contact between the metal substrate and the NaCl solution by restricting ion transport (Cl^–^, O_2_, H_2_O). However, if defects or permeation allow electrolyte penetration, electrochemical reactions occur at the metal-coating interface.

The anodic reaction involves metal dissolution, where Fe oxidizes to Fe^2^⁺and Fe^3+^ layers.

6 $${\text{Fe}} \to {\text{ Fe}}^{{{2} + }} + {\text{ 2e}}^{ - }$$

7 $${\text{Fe}}^{{{2} + }} \to {\text{Fe}}^{{{3} + }} + {\text{ e}}^{ - }$$

At the cathodic sites, oxygen reduction takes place, consuming the electrons released from the anodic reaction:

8 $${\text{O}}_{{2}} + {\text{4H}}^{ + } + {\text{4e}}^{ - } \to {\text{2H}}_{{2}} {\text{O}}$$

9 $${\text{O}}_{{{2} }} + {\text{2H}}_{{2}} {\text{O}} + {\text{4e}}^{ - } \to {\text{4OH}}^{ - }$$

Additionally, chloride ions (Cl^–^) promote the formation of soluble FeCl_2_, preventing the development of a stable protective oxide layer and thereby accelerating the corrosion process:

10 $${\text{Fe}}^{{{2} + }} + {\text{2Cl}}^{ - } \to {\text{FeCl}}_{{2}}$$

The incorporation of nAl_2_O_3_-Silane nanoparticles within the polymer matrix modifies these reactions by enhancing barrier properties, reducing ion permeability, and delaying both anodic and cathodic processes, thereby improving corrosion resistance and coating durability.

In contrast, coating without nanoparticles exhibit greater susceptibility to electrolyte penetration, leading to coating detachment due to the presence of microstructural pores in the polymer matrix. Furthermore, the improved protective performance is associated with the strong interfacial interactions between nAl_2_O_3_-Silane nanoparticles and the polymer matrix. The epoxidized terminal group of GMPTS can react with acrylate groups or hydroxyl groups in the oligomer during the UV curing process, contributing to an increase in crosslinking density. Additionally, the dispersion of nanoparticles in TPGDA promotes further network formation, along with the formation of Si–O–Al bonds^70^.

## Conclusion

Our study has demonstrated that incorporating nAl_2_O_3_-Silane nanoparticles into the ESOA@TMPTA polymer matrix yields a UV-polymer coating nanocomposite with enhanced mechanical, chemical, and corrosion resistance properties. Various characterization techniques, including FTIR, XRD, WCA, AFM, and SEM, confirmed the uniform dispersion of nanoparticles, homogeneity, and successful curing of the coatings.

The optimized formulation containing (8 wt%) of nAl_2_O_3_-Silane nanoparticles significantly improved key properties, including scratch resistance (39.2 N), gel fraction (97%), Gloss (95°), and chemical resistance compared to the neat ESOA@TMPTA polymer. The corrosion protection performance was also notably enhanced, as evidenced by EIS and PDP, where coatings with nAl_2_O_3_-Silane nanoparticles exhibited higher impedance values and lower corrosion current densities. The salt spray test in (5 wt%) NaCl solution for 500 h further confirmed the improved barrier properties. Additionally, the increase in contact angle values indicated surface hydrophobicity, contributing to enhanced corrosion resistance. The findings of this study contribute to advancing the development of UV-curable coatings, aligning with global sustainability efforts aimed at reducing energy consumption and volatile organic compound (VOC) emissions. This research not only provides insight into the design of high-performance protective coatings but also lays the foundation for future innovations in environmentally sustainable coating formulations. In addition, the final resultant coatings are transparent despite the presence of nAl_2_O_3_-silane nanoparticles. These findings differ from previous research in terms of transparency, as most polymer nanocomposites are translucent or opaque. According to our findings, the final coating can be effectively used to protect metal-based artifacts against corrosion since they provide a transparent coating layer.

## Data Availability

The datasets used and/or analysed during the current study available from the corresponding author on reasonable request.
